# De-Escalation of Broad-Spectrum and Last-Resort Antibiotics in Critically Ill Adults with Gram-Negative Infections: A Scoping Review and Evidence-Informed Framework for Tertiary-Care ICUs

**DOI:** 10.3390/antibiotics15090912

**Published:** 2026-09-16

**Authors:** Mihai Sava, Ioana Roxana Codru, Alina Simona Bereanu, Anca Maria Frățilă, Bogdan Ioan Vintilă

**Affiliations:** 1 County Clinical Emergency Hospital of Sibiu, 2–4 Corneliu Coposu Boulevard, 550245 Sibiu, Romania; mihai.sava@ulbsibiu.ro (M.S.); bogdan.vintila@ulbsibiu.ro (B.I.V.); 2 Faculty of Medicine, Lucian Blaga University of Sibiu, 2A Lucian Blaga Street, 550169 Sibiu, Romania; anca.fratila@ulbsibiu.ro

**Keywords:** antibiotic de-escalation, antimicrobial stewardship, critically ill, intensive care unit, Gram-negative bacteria, multidrug resistance, carbapenems

## Abstract

Background: Early broad-spectrum empirical therapy is life-saving in critical illness, but its unnecessary continuation drives resistance, toxicity, and cost; antibiotic de-escalation is the principal stewardship strategy for resolving this tension, yet its evidence base is fragmented and its practice inconsistent. This scoping review mapped the evidence on the definitions, timing, eligibility, implementation, safety, and clinical, microbiological, and resistance outcomes of de-escalating cephalosporins, carbapenems, colistin, and tigecycline in critically ill adults with suspected or confirmed Gram-negative infection. It translated this into a framework for a tertiary-care intensive care unit (ICU). Methods: We conducted a focused scoping review informed by JBI methodological guidance and reported according to PRISMA-ScR. The Web of Science Core Collection was searched for English-language publications from 1 January 2016 to 4 August 2026. Following deduplication, two reviewers independently screened titles and abstracts, and subsequently assessed potentially eligible full texts. Disagreements were resolved through discussion or consultation with a third reviewer. The review was designed to map the characteristics and range of the identified evidence rather than to provide an exhaustive systematic assessment or quantitative synthesis of intervention effects. (PROSPERO CRD420261478424). Results: We included 51 publications (35 empirical studies; 16 reviews, editorials, or consensus statements), with the empirical evidence being predominantly observational, including a single randomized trial. The definitions were heterogeneous, and the spectrum-ranking systems were non-uniform; reassessment typically occurred at 48–72 h. The reported de-escalation proportions ranged from approximately 10% in broadly defined treated populations to 71% in a selected, extractable ICU subgroup. These values were not directly comparable because studies used different definitions, eligibility criteria, time points, and denominators, including all patients treated with antibiotics, empirical-treatment episodes, microbiologically documented infections, and patients considered clinically eligible for de-escalation. Direct comparative studies did not identify a consistent increase in mortality following de-escalation; however, the predominantly observational evidence was vulnerable to confounding by indication, survivor bias, and treatment-selection bias, and did not establish equivalence, non-inferiority, or a survival benefit. Conclusions: De-escalation appears safe but rests on low-certainty, heterogeneous evidence. We propose an evidence-informed framework, a structured 48–72 h time-out, an eligible-patient denominator and a minimum monitoring dataset for tertiary ICUs, and identify standardized definitions and resistance-focused trials as research priorities. These components represent an evidence-informed implementation proposal developed by the authors and require prospective local validation.

## 1. Introduction

Antimicrobial de-escalation refers to narrowing or discontinuing broad-spectrum antibiotics based on clinical findings and microbiological data. This approach aims to reduce unnecessary antimicrobial exposure and treatment-related toxicity and is hypothesized to reduce ecological selection pressure. However, a reduction in the emergence of antimicrobial resistance has not been consistently demonstrated in clinical studies. Current international guidelines highlight the importance of assessing the need to de-escalate antibiotic therapy daily, rather than following a fixed treatment duration. A recent update reinforced this recommendation, stating that de-escalation should occur as soon as a confirmed microbiological diagnosis and susceptibility profile are available [1,2,3,4].

Antimicrobial resistance is one of the biggest threats to public health, and inappropriate or excessive antibiotic use is a major contributing factor. Choosing the right antibiotic treatment for critically ill patients is particularly challenging because the physiological changes associated with critical illness affect how drugs are absorbed, distributed, metabolized, and excreted [5,6,7]. The intensive care unit is the epicenter of antibiotic resistance. Patients in the ICU often undergo invasive procedures, experience extended hospital stays, and receive frequent broad-spectrum antibiotic treatments. As a result, they face a significantly higher risk of infections caused by multidrug-resistant Gram-negative bacteria, a problem that is particularly pressing in Eastern European ICUs [8,9,10,11,12].

Antibiotic de-escalation is not an isolated practice; rather, it reflects the effectiveness of a comprehensive stewardship system. The WHO Policy Guidance on Integrated Antimicrobial Stewardship Activities outlines stewardship as consisting of twelve interventions organized into five key pillars: national coordination and guidelines; access to and regulation of antimicrobials; awareness, education, and training; water, sanitation, and hygiene; and infection prevention and control, along with surveillance, monitoring, and evaluation. This approach intentionally extends stewardship beyond individual prescriptions to encompass the broader systems that facilitate effective antibiotic use [13].

Antibiotic de-escalation is a key antimicrobial stewardship strategy to reduce antibiotic overuse. Rapid molecular diagnostics can accelerate pathogen identification; however, access to these tests does not always lead to treatment changes. This uncertainty is even greater in intensive care units, where multidrug-resistant infections are common, and resources vary; effective alternative treatments may be limited, and practical, evidence-based guidance is often lacking [14,15,16].

However, despite its endorsement, evidence supporting de-escalation in critically ill patients is fragmented, and implementation remains inconsistent. No universally accepted definition of de-escalation currently exists. Different studies may include or exclude complete cessation of antibiotics and use different systems to rank the antibiotic spectrum. This variability limits comparability between studies and prevents meaningful data pooling [1,4,17]. In the prospective international DIANA cohort, antibiotic therapy was de-escalated in only 16% of patients within the first three days [18].

In this review, the term “last-resort antibiotics” is used operationally to denote agents reserved for severe Gram-negative infections when standard, narrower-spectrum, or less toxic alternatives are unavailable because of multidrug resistance, prior treatment failure, or patient-specific constraints. It primarily refers to colistin and, in selected multidrug-resistant infections, to tigecycline and carbapenems. Cephalosporins are included as broad-spectrum agents or as potential narrower, carbapenem-sparing alternatives and are not uniformly classified as last-resort antibiotics. Thus, “broad-spectrum” and “last-resort” are overlapping but not interchangeable categories within the evaluated de-escalation pathways.

To our knowledge, the available evidence has not been comprehensively mapped with respect to the definitions, timing, eligibility criteria, implementation strategies, safety outcomes, clinical and microbiological outcomes, and resistance consequences of de-escalation across the four target antibiotic classes in critically ill adults with Gram-negative infections. Accordingly, the clinical evidence-mapping objective and the local implementation objective were treated as distinct components of this work.

The four antibiotic classes were selected because they represent clinically important de-escalation decisions in ICUs with a high burden of multidrug-resistant Gram-negative organisms, even though their roles differ. Carbapenems are major targets of stewardship because of their broad spectrum and ecological impact; cephalosporins often serve as narrower or carbapenem-sparing alternatives when susceptibility permits; and colistin and tigecycline are Reserve or toxicity-limited agents whose continuation requires careful reassessment when safer, active alternatives are available. Other antipseudomonal β-lactams, β-lactam/β-lactamase inhibitor combinations, fluoroquinolones, aminoglycosides, and newer agents were not evaluated as independent class-specific targets unless they were part of a reported de-escalation pathway involving one of the four prespecified classes. This focused scope was chosen to maintain a clinically coherent review question rather than to provide an exhaustive review of all antibiotics used against Gram-negative infections.

The primary aim of this scoping review was to map and characterize the available evidence on antibiotic de-escalation with cephalosporins, carbapenems, colistin, and tigecycline in critically ill adults with suspected or confirmed Gram-negative bacterial infection. The secondary aim was to translate the mapped evidence into a preliminary, evidence-informed framework tailored to the epidemiological, microbiological, staffing, and resource constraints of Romanian tertiary-care ICUs. The proposed framework was not intended to constitute a formally graded clinical practice guideline and requires prospective local validation.

## 2. Results

### 2.1. Characteristics of the Included Evidence

The 51 included publications consisted of 35 empirical primary studies and 16 contextual sources. The contextual group encompassed evidence syntheses, reviews, guidelines, consensus documents, editorials, and viewpoints related to ICU antibiotic de-escalation. Most empirical primary studies were retrospective or prospective observational cohorts, before-and-after antimicrobial stewardship interventions, diagnostic-stewardship studies, and quality improvement programs; there was limited randomized evidence. Throughout the Results Section, “primary study” specifically refers to an empirical investigation with original data, while “publication” or “contextual source” refers to reviews, consensus statements, guidelines, editorials, and viewpoints. Contextual publications help interpret definitions, recommendations, and implementation principles and are not counted as standalone primary evidence for patient-level outcomes (Table 1).

The empirical studies included the reports by Kim, Sekandarzad, Lakbar, Yu, Mishima, Jover-Sáenz, Miller, Ali, Panditrao, Schumann, Gu, Zhu, Souza-Oliveira, Aissaoui, Roper, Rodríguez-Gómez, Aldardeer, Le, Arulappen, Trupka, Ghosh, Choudhuri, Contier, De Bus, Latorre Ibars, Kuwana, Kwon, Yoon, Ferrer, Salahuddin, Li, Niimura, Rodrigues, and Sellers and Nikolai [18,19,20,21,22,23,24,25,26,27,28,29,30,31,32,33,34,35,36,37,38,39,40,41,42,43,44,45,46,47,48,49,50,51,52]. The sample sizes ranged from 25 patients in a study on ESBL-producing Enterobacterales bacteremia [43] to 2730 ICU admissions in an antibiotic time-out study [23]. One hospital-wide stewardship study included 67,362 treated patients and reported ICU-specific antimicrobial-consumption outcomes [24].

The remaining 16 publications included narrative or structured reviews by Gardner, Ture, Giamarellou, Tanzarella, De Waele, Shirazi, Seok, Moniz, Mokrani, and Matuszak and Micek [17,54,55,56,58,59,60,61,62,63,64]; systematic reviews by Lakbar and Ohji [4,53]; an expert viewpoint by Benoit [65]; an editorial by Cortegiani [66]; and an expert consensus contribution [66]. These publications were used to contextualize definitions, implementation strategies, safety signals, and evidence gaps. We did not add patient numbers reported by these sources to the primary study populations because they substantially overlapped with the included empirical cohorts, particularly the DIANA study.

The studies originated from Europe, Asia, North and South America, North Africa, and the Middle East. Most were conducted in mixed medical–surgical, medical, surgical, neurological, or emergency ICUs. Specific populations included patients with sepsis or septic shock, ventilator-associated or hospital-acquired pneumonia, bloodstream infection, intra-abdominal infection, culture-negative infection, trauma, immunosuppression, and COVID-19.

### 2.2. Definitions and Timing of Antibiotic De-Escalation

Definitions varied substantially. Most studies defined de-escalation as replacing an empirical broad-spectrum antimicrobial with a narrower-spectrum agent, discontinuing one or more components of combination therapy, or both. Several studies included complete antibiotic discontinuation when infection was not microbiologically confirmed, whereas some reviews distinguished complete cessation from conventional de-escalation (Table 2).

Trupka et al. applied explicit antibiotic-spectrum hierarchies [38], as well as De Bus et al. [18], Roper et al. [33], Aldardeer et al. [35], Aissaoui et al. [32], and Nikolai et al. [52]. These systems classified de-escalation as a transition from a higher- to a lower-ranked agent. However, the rankings were not uniform: some were based primarily on antimicrobial spectrum, whereas others incorporated presumed ecological impact or WHO AWaRe categories. Consequently, transitions such as meropenem to cefepime or piperacillin–tazobactam to a third-generation cephalosporin were not necessarily classified the same way across all frameworks (Table 3).

Conventional reassessment was generally performed 48–72 h after empirical treatment initiation, as reported by Panditrao et al., Roper et al., De Bus et al., Le et al., and Aldardeer et al. [18,27,33,35,36]. Daily antibiotic review was implemented in the studies by Ali et al., Gu et al., and Trupka et al., and was consistently supported by the included reviews [26,29,38]. Aldardeer et al., Moniz et al., Matuszak et al., and Micek et al. recommended treatment modification within 24 h of definitive susceptibility results [35,61,63,64]. Pneumonia multiplex PCR provided results within approximately 1–4 h in the studies by Miller et al., Aissaoui et al., Zhu et al., Rodríguez-Gómez et al., and Contier et al.; however, result availability did not necessarily correspond to immediate antibiotic modification [25,30,32,34,41]. Daily reassessment was consistently supported across the narrative reviews, systematic reviews, and expert publications [17,26,29,38,58,60,61]. Several sources recommended that definitive narrowing occur within 24 h of obtaining reliable microbiological and susceptibility results.

### 2.3. Frequency of De-Escalation

Reported de-escalation rates ranged from approximately 10% to 71%. Comparability was limited by differences in population selection, eligibility criteria, and definitions.

Selected or protocol-driven populations reported higher rates. De-escalation occurred in approximately one-third of ICU patients treated during the COVID-19 period, approximately 48% in the study by Salahuddin [47], 67% of clinically eligible mechanically ventilated patients in the study by Trupka [38], and 71% of the extractable ICU subgroup in the study by Nikolai [52]. The Arulappen cohort included only patients who had undergone de-escalation and, therefore, could not be used to estimate its frequency [37].

Diagnostic-stewardship studies generally showed that rapid tests created opportunities to narrow therapy, but clinicians did not act on all actionable results. In Aissaoui et al., treatment was modified in 58% of mechanically ventilated patients after multiplex PCR, but only 11% underwent de-escalation or cessation [32]. An expert retrospective review identified another 26% in whom narrowing or cessation would have been appropriate. Similarly, rapid pneumonia PCR directly influenced 57.6% of treatment episodes in Rodríguez-Gómez et al., although de-escalation and escalation were not reported separately [34]. Miller et al. found no significant increase in overall de-escalation after panel implementation (71.2% versus 69.0%), although anti-MRSA therapy was discontinued earlier in a selected subgroup [25] (Table 4).

### 2.4. Findings According to Antibiotic Class (Table 5)

#### 2.4.1. Carbapenems

Carbapenems represented the most frequently evaluated predefined class. Ali et al. reported carbapenem de-escalation in 25.4% of eligible patients, without a difference in 30-day mortality. Sekandarzad et al. reported a reduction in meropenem exposure from 2.74 to 1.13 days of therapy [20]. Gu et al. observed a decrease in the proportion of carbapenem consumption from 23.07% to 14.43% [29]. However, Mokrani et al. found that only 14.9% of patients considered eligible for carbapenem de-escalation underwent the intervention [62].

**Table 5:** Antibiotic de-escalation by drug class: strategies, outcomes, and limitations of the current evidence.

Antibiotic Class	Main De-Escalation Strategies	Main Findings	Evidence Limitations
Carbapenems	Discontinuation; replacement with a susceptible narrower β-lactam; restriction through audit and feedback	Reduced meropenem/imipenem exposure; lower carbapenem consumption; no consistent increase in mortality or treatment failure	Mainly observational studies; variable eligibility and spectrum rankings
Cephalosporins	Transition from antipseudomonal or fourth-generation agents to third-generation or narrower cephalosporins; use as carbapenem-sparing therapy	Increased use of narrower cephalosporins in some stewardship programs; reduced fourth-generation cephalosporin exposure	Outcomes rarely stratified by cephalosporin generation
Colistin	Discontinuation or avoidance when microbiology supported a less toxic active agent	Colistin consumption decreased in one stewardship program but increased in another because of MDR Gram-negative infections	Very limited class-specific de-escalation and safety data
Tigecycline	Discontinuation or replacement when a narrower active option was available	Tigecycline or glycylcycline consumption decreased in several programs	No direct comparative study of tigecycline de-escalation

#### 2.4.2. Cephalosporins

Cephalosporins were commonly included in antibiotic-spectrum ranking systems and were frequently used as carbapenem-sparing alternatives. Jover-Sáenz et al. reported decreased fourth-generation cephalosporin consumption and increased third-generation cephalosporin consumption [24]. Kuwana et al. reported successful cefmetazole de-escalation in 11 patients with ESBL-producing Enterobacterales bacteremia [43]. All 11 survived. The small sample size and non-randomized design limited the inferences.

#### 2.4.3. Colistin and Tigecycline

Evidence for colistin- and tigecycline-specific de-escalation was insufficient for comparative effectiveness conclusions. Jover-Sáenz et al. reported decreased ICU use for colistin and glycylcyclines [24]. Conversely, Panditrao et al. observed increased colistin consumption, attributed to a high prevalence of MDR infections [27]. Yu et al. reported a numerical reduction in tigecycline use from 11.11% to 3.45%, but the difference was not statistically significant [22].

### 2.5. Mortality and Safety Outcomes

Mortality was reported across heterogeneous study designs and comparisons, including de-escalation versus no de-escalation, early versus late de-escalation, successful discontinuation versus treatment failure, acceptance versus rejection of stewardship recommendations, pre- versus post-intervention periods, and pooled secondary analyses. Most individual empirical studies reported no statistically significant difference in mortality between the comparison groups (Table 6).

The apparent reduction in mortality observed in some observational analyses should be interpreted cautiously, as de-escalation was more frequently performed in patients who had already demonstrated clinical improvement. Selection bias, survivor bias, and confounding by indication were acknowledged across the evidence base.

Safety outcomes were inconsistently measured. The pre-2016 randomized trial by Leone et al., summarized in several of the included reviews, reported longer total antibiotic treatment and a higher incidence of superinfection following de-escalation, with no significant difference in mortality [67]. Conversely, Roper et al. reported lower acute kidney injury incidence after de-escalation (10.5% vs. 27.4%) without increased Clostridioides difficile infection, hospital-acquired infection, or resistance emergence [33].

### 2.6. Clinical, Microbiological, and Resistance Outcomes

In Choudhuri et al., resistant organisms were detected less frequently following earlier than later de-escalation (9.7% vs. 25.8%) [40]. DIANA reported MDR emergence in 7.5% of patients with de-escalation and 11.9% of patients without de-escalation; the difference did not reach statistical significance. The systematic reviews concluded that the effect of de-escalation on subsequent resistance remains uncertain (Table 7).

### 2.7. Antibiotic Exposure and Length of Stay

Most intervention studies reported reductions in broad-spectrum antibiotic exposure. Sekandarzad et al. reduced overall antibiotic days of therapy from 12.95 to 9.91 days in critically ill patients with pneumonia [20]. Mishima et al. observed an immediate reduction in intravenous antibiotic days of therapy to 178.26 per 1000 patient-days after multidisciplinary antibiotic time-outs [23]. Roper et al. reported shorter pivotal antibiotic treatment (3 versus 6 days), and total antibiotic treatment (6 versus 8 days) following de-escalation [33]. However, reductions in broad-spectrum exposure did not invariably reduce total treatment duration. Aldardeer et al. found that broad-spectrum therapy was shorter after de-escalation (7.2 versus 10.3 days), while total antibiotic duration was similar [35]. The randomized study, incorporated into several reviews, reported a longer total treatment duration after de-escalation. This distinction between reducing the duration of broad-spectrum therapy and reducing the total duration of treatment was not consistently captured across studies. The pre-2016 randomized trial by Leone et al., cited in several included reviews, reported a longer total antibiotic-treatment duration following de-escalation (14.1 ± 13.4 vs. 9.9 ± 6.6 days). This finding highlights the distinction between reducing exposure to broad-spectrum agents and reducing the overall duration of antimicrobial therapy, which was not consistently reported across the included studies [67].

Length-of-stay findings were inconsistent. Roper et al. reported shorter ICU stays (5 versus 9 days) and hospital stays (9 versus 17 days) following de-escalation [33]. Niimura et al. reported a shorter stay among appropriately de-escalated patients with sepsis [49]. Conversely, Aldardeer et al. reported longer ICU and hospital stays in the de-escalation group, probably reflecting differences in patient selection, survival, and re-escalation [35]. DIANA and several pneumonia studies found no significant length-of-stay difference [18,20,28,38,44,48].

### 2.8. Factors Favoring De-Escalation

De-escalation was more likely when the initial empirical regimen was appropriate and microbiological assessment identified a susceptible organism for which a narrower active alternative was available (De Bus et al., 2020; Ali et al., 2022; Ghosh et al., 2023; Arulappen et al., 2025) [18,26,37,39]. Reliable cultures and susceptibility results, negative high-quality cultures, high-negative-predictive-value molecular tests, and the absence of relevant resistance determinants further supported treatment narrowing or discontinuation (Roper et al., 2023; Zhu et al., 2025; Aissaoui et al., 2025; Contier et al., 2025; Rodríguez-Gómez et al., 2025) [30,32,33,34,41]. Other favorable clinical factors included improvement or hemodynamic stability, decreasing organ dysfunction or biomarker concentrations, adequate source control, monomicrobial infection, and the absence of an uncontrolled concurrent infectious focus (De Bus et al., 2020; Trupka et al., 2017; Ghosh et al., 2023; Le et al., 2024; Arulappen et al., 2025) [18,36,37,38,39]. Organizational facilitators included daily multidisciplinary reassessment, pharmacist and microbiologist participation, formal antibiotic time-outs at 48–72 h, local antibiograms, and institutional stewardship protocols (Trupka et al., 2017; Panditrao et al., 2021; Ali et al., 2022; Gu et al., 2023; Mishima et al., 2023) [23,26,27,29,38].

Conversely, septic shock, persistent hemodynamic instability, worsening organ dysfunction, uncertain or inadequate source control, polymicrobial infection, previous antibiotic exposure, and concurrent infectious foci reduced the likelihood of de-escalation (Trupka et al., 2017; De Bus et al., 2020; Choudhuri et al., 2021; Roper et al., 2023; Arulappen et al., 2025) [18,33,37,38,40]. Negative, unreliable, or unavailable cultures also represented important barriers because they limited the identification of an appropriate narrower regimen (Lakbar et al., 2020; Roper et al., 2023; Le et al., 2024) [4,33,36]. The isolation or suspected presence of MDR and extensively drug-resistant or carbapenem-resistant organisms frequently discouraged narrowing, particularly when no narrower active alternative was available (Souza-Oliveira et al., 2016; Aldardeer et al., 2023; Zhu et al., 2025) [30,31,35]. Rapid molecular diagnostics facilitated de-escalation when negative results had a high negative predictive value; however, their clinical application was limited by concerns regarding false-positive detections, colonization, residual microbial DNA, incomplete resistance-marker coverage, and off-panel pathogens (Aissaoui et al., 2025; Rodríguez-Gómez et al., 2025; Contier et al., 2025; Yoon et al., 2026) [32,34,41,45]. Positive microbiological findings, therefore, had a context-dependent effect: they supported narrowing when a susceptible pathogen and an active lower-spectrum alternative were identified but discouraged de-escalation when they demonstrated ESBL-producing, carbapenem-resistant, or extensively drug-resistant organisms (Kuwana et al., 2020; Aldardeer et al., 2023; Ghosh et al., 2023) [35,39,43]. Finally, clinician concern about treatment failure, inadequate stewardship staffing, lack of institutional protocols, and delayed communication of microbiological results constituted recurrent implementation barriers (Panditrao et al., 2021; Miller et al., 2023; Roper et al., 2023; Aissaoui et al., 2025) [25,27,32,33] (Table 8).

### 2.9. Policy- and System-Level Findings

The included contextual and stewardship publications identified several recurring policy- and system-level requirements for effective de-escalation. First, de-escalation should be embedded within institutional antimicrobial-stewardship governance rather than implemented as an isolated prescribing decision. Relevant components included locally adapted empirical treatment guidelines, an ICU-specific antibiogram, standardized microbiological sampling, timely reporting of susceptibility results, and a formal 48–72 h antibiotic review. Second, implementation depended on access to microbiology, infectious-disease, pharmacy, and critical-care expertise, as well as the availability of narrower, microbiologically active alternatives. Third, prospective audit and feedback, clinician education, documentation of the reason for continuing broad-spectrum treatment, and regular reporting of antimicrobial use were recurrently identified as implementation supports. Finally, stewardship policies were linked to infection-prevention and surveillance activities because de-escalation opportunities depend on local resistance epidemiology and the reliability of microbiological information [13,60,63,68,69].

These findings primarily originate from stewardship interventions and reviews, policy guidance, and consensus publications. They should be seen as implementation principles rather than direct evidence that a specific policy alone enhances patient outcomes.

### 2.10. The Proposed Evidence-to-Practice Framework for a Romanian Tertiary ICU

The evidence supports a structured, resource-conscious de-escalation program centered on microbiological sampling, clinical reassessment, and multidisciplinary decision-making. For a Romanian tertiary hospital with a high expected burden of MDR Gram-negative organisms, an ICU-specific antibiogram, standardized microbiological sampling, a formal antibiotic time-out at 48–72 h, daily prescription review, and a prospective audit with feedback should be prioritized. Rapid molecular diagnostics should be selectively rather than universally introduced, because their clinical benefit depends on appropriate patient selection, conventional culture confirmation, and clinicians’ ability to act promptly on results (Table 9).

### 2.11. Standard 48–72 H Antibiotic Time-Out

**Figure 1:**
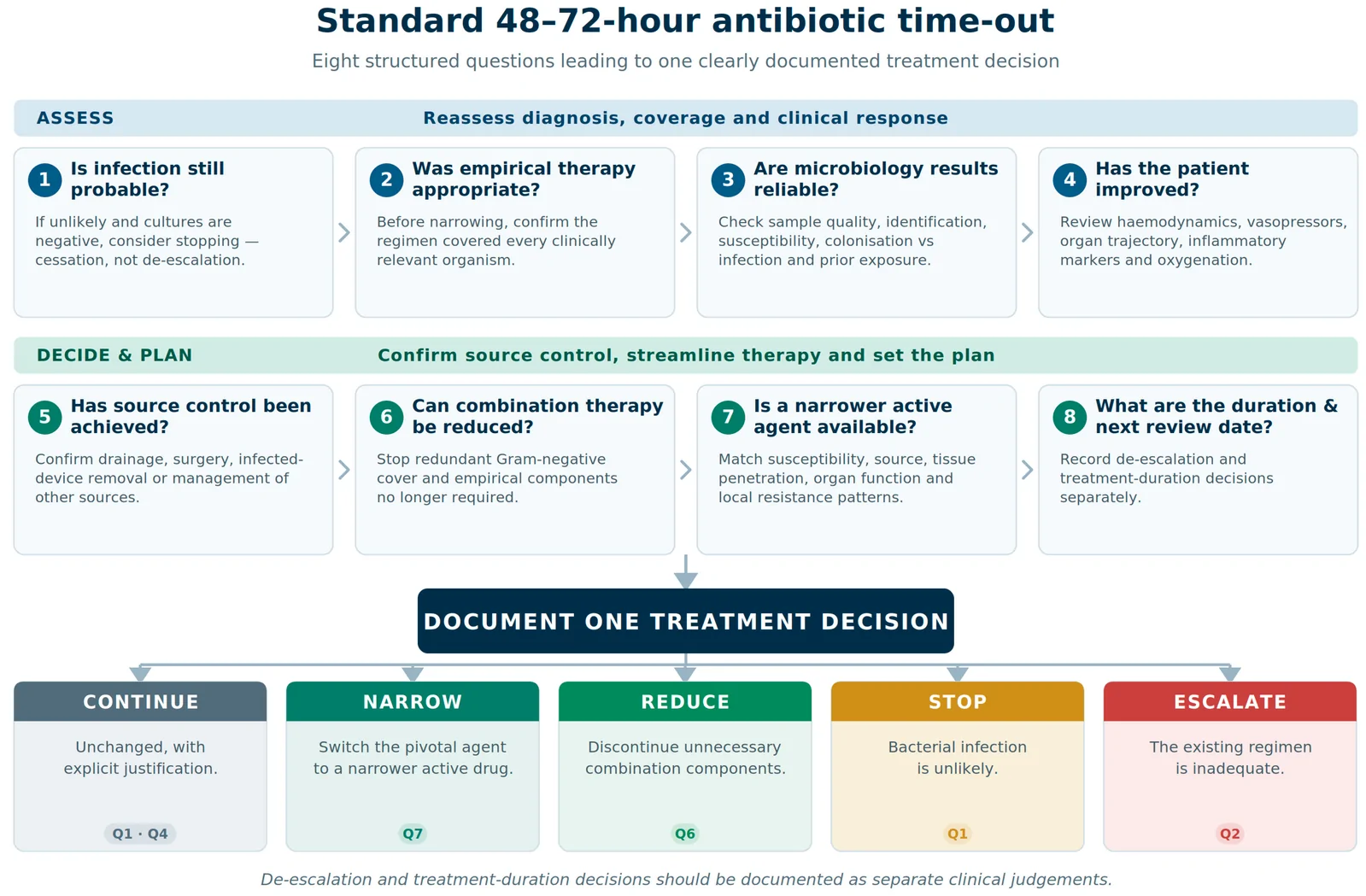
The proposed structured 48–72 h antibiotic time-out for the critically ill. Eight sequential questions guide the team to one clearly documented decision (continue, narrow, reduce, stop, or escalate), with de-escalation and treatment-duration judgments recorded separately. Conceptual figure developed by the authors and drafted with the assistance of generative AI.

At each formal reassessment, the clinical team should document the following (Figure 1):

1. Is bacterial infection still probable? If infection is unlikely and adequate samples are negative, consider antibiotic cessation rather than classifying the decision as conventional de-escalation.
2. Was the initial empirical regimen appropriate? Confirm that the regimen covers all clinically relevant organisms before narrowing treatment.
3. Are microbiological results reliable? Review sample quality, organism identification, susceptibility results, possible colonization or contamination, and previous antimicrobial exposure.
4. Has the patient improved? Assess hemodynamic stability, vasopressor requirements, organ dysfunction trajectory, inflammatory markers, and oxygenation.
5. Has adequate source control been achieved? Confirm drainage, surgery, removal of infected devices, or management of other relevant sources.
6. Can combination treatment be reduced? Discontinue redundant Gram-negative coverage and other empirical components that are no longer required.
7. Is a narrower active agent available? Select definitive therapy according to susceptibility, infection source, tissue penetration, organ function, and local resistance patterns.
8. What is the planned duration and next review date? Record de-escalation and treatment-duration decisions separately.

### 2.12. Suggested Documentation Outcome

Each time-out should result in one of five documented decisions:

- Continue unchanged, with justification;
- Narrow the pivotal antibiotic;
- Discontinue unnecessary combination components;
- Stop antibiotics because bacterial infection is unlikely;

Escalate because the existing regimen is inadequate. The strongest evidence supports carbapenem and broad-spectrum β-lactam de-escalation. Cephalosporins were frequently used as carbapenem-sparing or lower-ranked agents, although the choice should be based on microbiological and clinical suitability rather than spectrum ranking alone. Evidence supporting class-specific de-escalation of colistin and tigecycline remains insufficient; therefore, recommendations for these agents should emphasize mandatory reassessment, avoidance of unnecessary continuation, and replacement with a safer active alternative when available rather than prescriptive switching rules (Table 10).

### 2.13. Minimum Dataset for Local Monitoring

A Romanian tertiary ICU should not assess program success solely by total antibiotic consumption. The following indicators would provide a more balanced evaluation, as shown in Table 11.

The denominator for the de-escalation rate should be patients eligible for de-escalation, rather than all of the patients receiving antibiotics. Eligibility should require an adequate initial regimen, clinical stability or improvement, interpretable microbiological information, adequate source control, and the availability of an active narrower alternative. This approach reduces misleading comparisons between ICUs caring for populations with different illness severity and resistance profiles.

## 3. Discussion

The present focused scoping review mapped 51 publications on de-escalation of broad-spectrum and last-resort antibiotics in critically ill adults with suspected or confirmed Gram-negative infections. For interpretation, direct patient-level de-escalation studies were distinguished from broader stewardship interventions, diagnostic or antibiotic time-out studies, and secondary or contextual publications. The identified evidence was then translated into a preliminary evidence-informed framework for Romanian tertiary-care ICUs. This framework should be regarded as an implementation proposal requiring prospective validation rather than as a formally developed or validated clinical practice guideline.

Among the empirical primary studies, the evidence was predominantly observational, before-and-after, and single-center, while randomized evidence was limited. The remaining publications provided secondary or contextual evidence concerning definitions, recommendations, stewardship implementation, and diagnostic support. These different categories were not considered equivalent sources of evidence for patient-level effectiveness. Both Tabah and colleagues [1] and Lakbar and colleagues [4,21] noted that the evidence was of low certainty and predominantly non-randomized. Additionally, a Cochrane review reported too few randomized trials to reliably assess safety or efficacy [70]. The single large prospective cohort study, DIANA [18], served as the reference dataset. Therefore, Table 1 sets a crucial expectation for the reader: interpret every subsequent finding within the context of uncertainty imposed by the study designs, with confounding by indication as the primary risk throughout.

Weiss and colleagues [71] proposed a consensus definition that permits ranking β-lactams precisely because expert panels disagreed, and the 2020 ESICM/ESCMID [72] position statement was convened in part to standardize terminology; yet heterogeneity persists in primary studies. The most consistent finding across studies in this review was definitional heterogeneity. Researchers used various criteria to count transitions, such as spectrum narrowing, discontinuation of redundant combination components, conversion to monotherapy, or complete cessation [18,21,38]. They also applied non-uniform ranking systems for these transitions, leading to inconsistent classification. This reflects a long-standing concern raised by Tabah and colleagues [72] and reiterated in subsequent systematic reviews. As a result, outcomes cannot be meaningfully pooled, and any guideline recommendation carries the ambiguity of its underlying definitions. To improve comparability, it is essential to standardize the operational definition and, in particular, to reach a consensus on whether complete cessation is considered a form of de-escalation or a distinct action. In our review, we addressed this issue by extracting cessation data separately.

Antibiotic spectrum ranking systems used to classify de-escalation vary in their approaches. They can be based on the antimicrobial spectrum, presumed ecological impact, or WHO AWaRe categories [18,21,38,73,74]. The literature offers several direct solutions that have not been widely adopted. These include the Weiss β-lactam ranking, the spectrum-score methods developed by Madaras-Kelly and Moehring, the antibiotic spectrum index, and the more recent Simplified Spectrum Score [68,71,75,76,77]. These tools help convert subjective judgments into objective, auditable measures, which would significantly enhance comparability between studies and among intensive care units. This analysis supports a clear recommendation: future de-escalation research and local monitoring should use a single validated spectrum score instead of custom rankings.

Reassessment clustered at 48–72 h, with several sources recommending definitive narrowing within 24 h of reliable susceptibilities, the threshold endorsed by the ESICM/ESCMID statement [72]; yet, the reported frequency of de-escalation spanned roughly 10% to 71%, the highest figures arising in selected or protocol-driven populations and the lowest where denominators included all treated patients [31,52,78]. This wide range is consistent with the broader literature, in which DIANA [18] de-escalated only 16% of patients within three days, and earlier syntheses describe comparable variation driven by denominator and definition rather than by true differences in practice. Two messages recur. First, de-escalation is frequently not performed even when it appears microbiologically feasible: rapid molecular diagnostics shortened pathogen identification and reliably excluded pathogens through high negative predictive values, but their availability did not translate into treatment change [34], a negative Gram-negative result rarely prompted carbapenem discontinuation, and only a minority of actionable panels led to narrowing. Diagnostics, in other words, are enablers, not decisions; their value is realized only when every result is tied to a stewardship recommendation and a documented action, which is why our framework couples selective rapid testing with a mandatory time-out and audit-and-feedback rather than deploying diagnostics in isolation. Second, comparisons of raw rates are misleading unless the denominator is standardized to eligible patients, the rationale for the eligible-patient denominator we propose.

The evidence was markedly asymmetric across the four target classes: carbapenems were the best-studied, with consistent reductions in exposure through stewardship and no consistent mortality penalty, while cephalosporins featured chiefly as carbapenem-sparing or lower-ranked alternatives, and colistin- and tigecycline-specific de-escalation evidence was sparse and contradictory, consumption falling in some programs and rising in others, in line with local multidrug-resistant epidemiology [24,27,29,43,62]. This mirrors the literature’s concentration on carbapenem stewardship and carbapenem-sparing strategies for extended-spectrum β-lactamase producers, an area where caution is nonetheless warranted, given trial signals against some sparing choices for bloodstream infection. For colistin and tigecycline, the scarcity of de-escalation data, together with their Reserve status in the WHO AWaRe framework, their narrow therapeutic windows, and colistin’s recognized nephrotoxicity, supports the position taken in our class-specific recommendations: mandatory specialist reassessment and replacement with a safer active agent where available, rather than prescriptive switching rules.

Across the direct comparative studies, de-escalation was not consistently associated with increased mortality. This finding should be interpreted as the absence of a reproducible mortality penalty within the identified evidence rather than as proof of equivalence, non-inferiority, or survival benefit. Most estimates were derived from observational studies in which de-escalation was more likely to be performed in patients who had already improved clinically. Confounding by indication, survivor bias, and treatment-selection bias may, therefore, explain some of the apparent benefit reported in observational comparisons. Randomized evidence remained limited. A sepsis meta-analysis found no detrimental effect, the single randomized trial [67] found no mortality difference, and DIANA [18] found no penalty alongside a higher day-7 clinical cure, consistent too with the ESICM/ESCMID [72] position statement. The apparent survival advantage in observational analyses should not be read as a treatment effect; however, de-escalation is preferentially applied to patients already improving, so confounding by indication, survivor bias, and selection bias inflate any benefit, as we and the source studies acknowledge. The Leone trial [67] is instructive in the opposite direction, finding no mortality difference but a longer total treatment duration and more superinfections after de-escalation, underlining, first, that reducing the breadth of therapy is not the same as reducing its duration, and that the two should be evaluated and reported as distinct interventions. We should, therefore, read the information as evidence of safety with respect to mortality, not of a survival benefit. Findings from general antimicrobial-stewardship programs, rapid-diagnostic interventions, antibiotic time-outs, and analyses comparing acceptance versus rejection of stewardship recommendations were interpreted as implementation evidence rather than as direct estimates of the clinical effect of de-escalation. Their outcomes may reflect several simultaneous changes in prescribing practices, diagnostic turnaround times, multidisciplinary review, or institutional organization and cannot be attributed exclusively to antibiotic de-escalation. Secondary reviews and consensus publications were used to contextualize the primary evidence and were not counted as independent patient-level outcome data.

**Figure 2:**
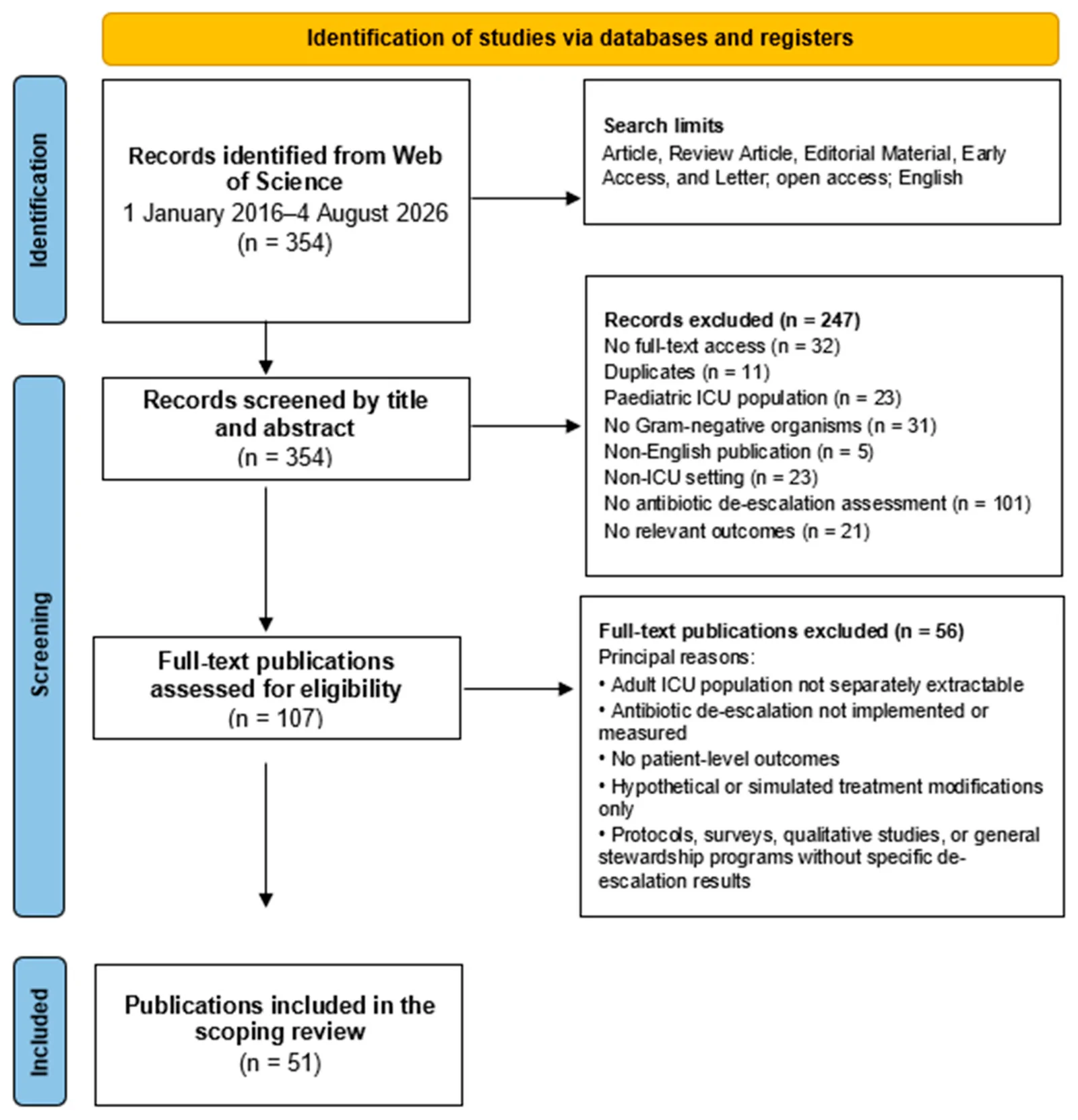
The flow of publications through the scoping review selection process.

Positioned within the WHO five-pillar [79] model of integrated stewardship, de-escalation is best understood as the bedside expression of a functioning stewardship system rather than a stand-alone act: it depends on local guidelines, a supply chain that stocks narrower alternatives, clinician education, infection prevention and reliable microbiology, and surveillance with audit-and-feedback. Table 9 translates this into a resource-conscious program, an ICU-specific antibiogram, standardized sampling, a formal 48–72 h time-out (with the decision logic made explicit in Figure 2, which ties each reassessment to one documented outcome), daily multidisciplinary review, prospective audit-and-feedback, and selective rather than universal rapid diagnostics—each element corresponding to a component of established stewardship guidance. Its contribution is contextual specificity: whereas most stewardship frameworks are generic, this one is calibrated to a high-multidrug-resistant, resource-variable tertiary ICU, such as a Romanian center, a setting with among the highest multidrug-resistant Gram-negative burdens in Europe [80]. Recent single-center surveillance underscores that narrower alternatives may be scarce and rapid diagnostics costly. Reporting the de-escalation rate against a denominator of eligible patients, rather than all treated patients, is essential to avoid penalizing units that care for sicker, more resistant populations.

The feasibility of this framework in Romanian ICUs must be interpreted in relation to local resistance epidemiology and microbiological capacity. Recent Romanian ICU surveillance has documented a substantial burden of multidrug-resistant and carbapenem-non-susceptible Gram-negative organisms, particularly *Acinetobacter baumannii* [11,12,73,80,81]. In such settings, a narrower microbiologically active alternative may not be available even after organism identification and susceptibility testing. Delays in pathogen identification, susceptibility reporting, or communication of actionable microbiological results may also prolong empirical broad-spectrum therapy or delay the transition to definitive active treatment [15,69,81,82]. These constraints reduce the proportion of patients who are genuinely eligible for de-escalation and support the use of ICU-specific antibiograms, standardized microbiological sampling, rapid communication of critical results, and an eligible-patient denominator for local audit. These Romanian data provide contextual support for the proposed framework but do not directly demonstrate the safety or effectiveness of de-escalation. The framework should not be interpreted as a set of Romanian-specific effectiveness recommendations. Its clinical decision principles are based on international evidence, while its proposed implementation pathway and monitoring indicators were adapted to the resistance epidemiology and resource constraints of Romanian tertiary-care ICUs.

Narrowing does not necessarily ensure adequate pharmacologic coverage. Critically ill patients often have altered antimicrobial pharmacokinetics, and a narrower, microbiologically active agent may still provide inadequate exposure with conventional dosing. De-escalation decisions should, therefore, incorporate renal function, augmented renal clearance, renal-replacement or extracorporeal support, infection site, minimum inhibitory concentration, and the feasibility of prolonged or continuous β-lactam infusion. When available locally, therapeutic drug monitoring may complement, not replace, microbiological and clinical reassessment, particularly in patients with marked pharmacokinetic variability [83].

De-escalation or discontinuation at the end of life represents a distinct stewardship and goal-concordant-care context. Findings from this population primarily concern avoidance of non-beneficial treatment and should not be extrapolated to the clinical effectiveness of de-escalation in patients receiving active curative treatment [19].

### 3.1. Limitations

This review has a key limitation related to its information sources. The search was limited to the Web of Science Core Collection and was not conducted in other databases. As a result, publications indexed exclusively in these other databases might have been overlooked, meaning this review should not be considered a complete systematic evaluation of all clinical evidence on antibiotic de-escalation. This restriction could particularly impact the retrieval of regional journals, recently added publications, and clinical studies with limited citation visibility. Additionally, eligibility was limited to English-language publications from 2016 onward. These choices enhanced the review’s feasibility and focus on current clinical practice but at the cost of narrowing the overall breadth of the evidence included. The definitions were heterogeneous, the resistance and ecological outcomes were rarely measured, and colistin- and tigecycline-specific data were too sparse for class-specific conclusions. Finally, the proposed framework is an evidence-informed decision aid, not a validated guideline, and its prospective performance in a Romanian tertiary ICU remains to be demonstrated.

### 3.2. Future Directions

These gaps define the priorities: a standardized operational definition specifying how complete cessation is handled; adequately powered trials reporting resistance and ecological outcomes (not mortality alone) and separating spectrum reduction from duration reduction; consistent reporting against an eligible-patient denominator; and prospective evaluation of implementation bundles, including the framework, time-out, and minimum dataset proposed here, in high-multidrug-resistant, resource-variable ICUs, such as those in Romania.

## 4. Materials and Methods

### 4.1. Review Design and Reporting Framework

This scoping review mapped the available evidence on antibiotic de-escalation in critically ill adults receiving broad-spectrum or last-resort antibiotics for suspected or confirmed Gram-negative bacterial infections. We selected a scoping-review design because of expected heterogeneity in study designs, patient populations, de-escalation definitions, antimicrobial regimens, reassessment time points, and reported outcomes.

The review design followed JBI methodological guidance for scoping reviews and was reported following the PRISMA-ScR guidelines. PRISMA-ScR provided a framework to ensure a transparent account of the review question, eligibility criteria, sources, selection process, data charting, and synthesis. Since the search was limited to one bibliographic database, this review is considered a focused evidence map rather than a comprehensive systematic review of all of the relevant clinical literature. Search methods were detailed following the relevant PRISMA-S items. We will provide the PRISMA-ScR checklist as Supplementary Material.

The protocol was submitted to PROSPERO before screening began and was formally registered on 14 August 2026 under registration number CRD420261478424, while article selection was ongoing. Accordingly, although the protocol was developed and submitted before screening, the registration was not fully prospective. There were deviations from the protocol.

### 4.2. Review Question and PCC Framework

This review followed the Population–Concept–Context framework. The population included critically ill adults admitted to an ICU or comparable critical-care environment and treated for suspected or microbiologically confirmed Gram-negative bacterial infection. The principal concept was antibiotic de-escalation involving cephalosporins, carbapenems, colistin, or tigecycline. The relevant strategies included spectrum narrowing, discontinuation of unnecessary components of combination therapy, conversion from combination therapy to active monotherapy, carbapenem-sparing treatment, and replacement of colistin or tigecycline with a safer or more targeted active agent. The context comprised adult ICUs and comparable critical-care settings, with particular attention to tertiary hospitals and healthcare systems with a high prevalence of multidrug-resistant Gram-negative organisms.

The primary review question was: What evidence is available on the definitions, timing, eligibility criteria, implementation strategies, safety, clinical and microbiological outcomes, and resource requirements for de-escalating cephalosporins, carbapenems, colistin, and tigecycline in critically ill adults with suspected or confirmed Gram-negative bacterial infections? Secondary questions addressed factors favoring de-escalation, barriers to implementation, and the applicability of the identified strategies to a Romanian tertiary-care ICU.

### 4.3. Operational Definition of Antibiotic De-Escalation

For this review, antibiotic de-escalation was operationally defined as one or more of the following changes after reassessment of empirical or initial antibiotic treatment:

- The replacement of a broad-spectrum antibiotic with an agent having a narrower antibacterial spectrum;
- The discontinuation of one or more unnecessary components of combination therapy;
- Conversion from combination therapy to microbiologically active monotherapy;
- The replacement of a carbapenem with a susceptible carbapenem-sparing agent;
- The replacement of colistin or tigecycline with a safer or more targeted active agent;
- Pathogen- and susceptibility-directed reduction in the ecological impact of treatment.

Complete discontinuation of antibiotics when bacterial infection was considered unlikely or excluded was extracted separately as “antibiotic cessation.” Because some definitions do not consider complete cessation as representative of classical de-escalation, it was not automatically combined with spectrum-narrowing interventions. Dose adjustment, route optimization, therapeutic drug monitoring, and shortening of treatment duration were not considered de-escalation when performed in isolation. These interventions were nevertheless recorded when they formed part of a broader de-escalation or stewardship strategy.

In this review, the term “last-resort antibiotic” was considered an operational clinical designation rather than a strict regulatory category. It referred to drugs used for severe Gram-negative infections when narrower, less toxic, or more conventional treatments were inaccessible due to multidrug resistance or other clinical factors. Typically, this classification mainly included colistin and, depending on the organism, susceptibility, infection site, and available options, extended to tigecycline and carbapenems. Cephalosporins, regarded as broad-spectrum or as alternatives to carbapenems, were not consistently categorized as last-resort antibiotics.

### 4.4. Information Source and Search Strategy

The Web of Science Core Collection was chosen as the source of bibliographic data because this review covers critical care, infectious diseases, clinical microbiology, pharmacology, and antimicrobial stewardship. Its multidisciplinary nature and citation indexing made it appropriate for this review’s goal of descriptive evidence mapping. The search aimed to identify and characterize various definitions, implementation methods, clinical criteria, reported outcomes, and evidence gaps related to antibiotic de-escalation, rather than estimating a pooled effect of interventions.

The database was searched for publications from 1 January 2016 to 4 August 2026. The search combined terms related to antibiotic or antimicrobial de-escalation, intensive care or critical illness, Gram-negative infection, antimicrobial stewardship, and relevant antibiotic classes. It was limited to English-language publications and specific document types: articles, review articles, editorial materials, early access, and letters. The initial search yielded 354 records before screening.

The full, reproducible search strategy, including the Web of Science collection used, search strings, Boolean operators, field tags, date and language restrictions, document-type limits, execution date, and number of records retrieved, is detailed in the PRISMA Check List in the Supplementary Materials. Strict reliance on a single database was a predefined methodological limitation and was considered when interpreting the completeness and generalizability of the evidence map.

### 4.5. Eligibility Criteria

Population and setting: Studies were eligible if they included adults aged 18 years or older admitted to an ICU or comparable critical-care setting. Studies involving mixed hospital populations were included only when ICU data could be extracted separately. Studies limited to pediatric or neonatal ICUs, conventional wards, emergency departments, hematology units, or other non-critical-care settings were excluded unless a distinct ICU subgroup was reported.

Infection characteristics: Eligible studies investigated suspected or confirmed Gram-negative bacterial infection, including sepsis or septic shock, bloodstream infection, hospital-acquired or ventilator-associated pneumonia, urinary tract infection, intra-abdominal infection, and other severe infections encountered in critical care. Studies of colonization without suspected infection and studies evaluating antimicrobial prophylaxis were excluded.

Antimicrobial exposure: Studies were required to evaluate at least one of the following antibiotic classes: cephalosporins, carbapenems, and polymyxins, including colistin, or tigecycline. Broad ICU de-escalation studies were also eligible when one or more target classes formed part of the empirical regimen, even if outcomes were not reported separately for every antibiotic class.

Concept and outcomes: Eligible primary studies had to describe an identifiable de-escalation strategy, an opportunity for de-escalation, or objective criteria used to support de-escalation. Relevant outcomes included: frequency and timing of de-escalation; mortality; clinical cure or treatment failure; relapse, recurrence, reinfection or superinfection; microbiological eradication; emergence or selection of antimicrobial resistance; re-escalation of treatment; adverse drug effects and nephrotoxicity; antibiotic exposure or consumption; ICU or hospital length of stay; costs and resource utilization; factors favoring de-escalation; and barriers or reasons for continuing broad-spectrum treatment. Studies examining only pharmacokinetics, dosing, treatment duration, or antibiotic consumption without an identifiable de-escalation component were excluded from the primary evidence synthesis.

Sources of evidence: Eligible studies included randomized trials, cohort, case-control, before-and-after studies, interrupted time series, and antimicrobial stewardship interventions. Systematic reviews, guidelines, consensus statements, and expert papers were used as contextual sources for ICU de-escalation, summarized separately and not replacing primary evidence. Excluded were protocols without results, simulations without patient data, editorials without evidence, and isolated case reports. Only English articles from 1 January 2016 onward were included, reflecting current ICU epidemiology, diagnostics, resistance, and stewardship practices.

### 4.6. Study Selection

All records were exported to Mendeley Reference Manager, and duplicates were removed electronically and confirmed manually. Screening was performed in two stages. First, titles and abstracts were assessed against the predefined eligibility criteria. Records considered potentially relevant or insufficiently described in the abstract proceeded to full-text assessment. Second, full texts were evaluated against the complete eligibility criteria. The reasons for exclusion at the full-text stage were recorded using standardized categories, including non-ICU populations; ICU results not separately extractable; pediatric or neonatal population; absence of Gram-negative infection; no identifiable de-escalation strategy; no relevant antibiotic exposure; no relevant outcomes; protocol without results; simulation or hypothetical intervention; duplicate publication; full text unavailable; and language outside the eligibility criteria.

The screening was conducted independently by IRC and BIV. We resolved disagreements through discussion and, when needed, consultation with a third reviewer, MS. We present the selection process using a PRISMA flow diagram (Figure 1). We linked multiple publications reporting the same population and treated them as a single source of evidence unless they reported distinct outcomes. Final consensus decisions were not used to calculate a retrospective kappa statistic because they no longer represented the reviewers’ initial independent assessments.

### 4.7. Data Charting

We developed a standardized data-charting form in Microsoft Excel and piloted it on the 51 eligible publications, comprising 35 empirical primary studies and 16 contextual sources. The form was refined iteratively when additional relevant variables were identified. Data charting included: first author and publication year; country; study design and setting; sample size and population; infection source; microorganisms and resistance phenotypes; initial antibiotic regimen; antibiotic class studied; definition of de-escalation; de-escalation strategy; timing of reassessment; clinical, microbiological and biomarker criteria; primary outcome; mortality; clinical and microbiological outcomes; resistance outcomes; factors favoring de-escalation; barriers or reasons against de-escalation; authors’ conclusions; and study limitations. The data were charted by IRC using the standardized form. BIV independently verified each data field for every included primary study and contextual source against the corresponding full-text publication. Verification covered the study design, population, sample size, intervention and comparator definitions, de-escalation criteria, numerical outcomes, effect estimates, author attribution, and reference number. Discrepancies were recorded in the data-charting file, discussed by the two reviewers, and resolved by re-examining the original publication. Unresolved disagreements were referred to MS for adjudication. The final entry was accepted only after reviewer agreement. For reviews and consensus documents, we charted only ICU-relevant evidence and recommendations under the authorship of the secondary source. We did not count outcomes from primary studies cited by these publications twice when the corresponding primary study was independently included.

### 4.8. Evidence Synthesis

We summarized the charted evidence descriptively and narratively. We reported numerical outcomes using the effect estimates provided by the original studies. We did not plan a meta-analysis because we anticipated heterogeneity in populations, de-escalation definitions, comparator groups, timing, and outcome measurement. We tabulated definitions and reassessment time points to demonstrate methodological heterogeneity. Safety outcomes were interpreted cautiously because observational de-escalation studies are particularly susceptible to selection, indication, and survivor bias: patients who improve are more likely to undergo de-escalation than patients with persistent instability.

Conflicting findings were compared according to the directness of the evidence, study design, patient population, infection type, de-escalation definition, eligibility denominator, timing of reassessment, comparator, and outcome definition. Direct patient-level comparative studies were prioritized when interpreting mortality, clinical cure, recurrence, and safety. Broader stewardship and diagnostic studies were interpreted primarily as implementation evidence because their effects could not be attributed exclusively to de-escalation. When studies reported discordant findings, we examined whether differences could be explained by patient selection, illness severity, microbiological documentation, intervention timing, or confounding by indication. Findings were presented narratively in their original direction and magnitude; no vote-counting rule or pooled estimate was applied.

### 4.9. Critical Appraisal of Individual Sources of Evidence

No formal risk-of-bias or methodological-quality instrument was applied. This decision reflected this review’s scoping and evidence-mapping purpose: to characterize the range, definitions, implementation approaches, outcomes, and gaps in the available literature, rather than to estimate a pooled treatment effect or establish comparative effectiveness. The included publications represented heterogeneous evidence types for which no single appraisal instrument was applicable, including randomized and observational studies, before-and-after interventions, diagnostic and stewardship studies, reviews, consensus documents, and contextual publications.

Methodological characteristics relevant to interpretation—including the study design, comparator, population, sample size, limitations, and susceptibility to confounding—were nevertheless charted systematically. Findings from observational comparisons were interpreted cautiously because de-escalation is particularly vulnerable to confounding by indication, survivor bias, and treatment-selection bias. The absence of formal risk-of-bias assessment means that the findings should be interpreted as a map of the identified evidence rather than as formally graded evidence of effectiveness.

### 4.10. The Development of the Romanian Tertiary-ICU Framework

The practical framework was developed from the charted evidence through an evidence-to-practice mapping process. Recommendations were formulated only when supported by at least one of the following: direct comparative evidence in critically ill adults; consistent findings across several ICU observational studies; ICU-specific consensus supported by compatible primary evidence; and reproducible diagnostic or stewardship interventions with demonstrated effects on antibiotic use.

The framework was developed through a structured evidence-to-practice mapping process. First, charted findings were organized into predefined clinical and implementation domains: probability of ongoing bacterial infection; adequacy of empirical treatment; reliability of microbiological findings; clinical stability; source control; combination-therapy reduction; availability of a narrower active agent; timing and duration; staffing and diagnostic resources; and monitoring outcomes.

Second, candidate recommendations were linked to their supporting evidence category. Greater interpretive weight was given to direct comparative evidence in critically ill adults, followed by consistent findings across several observational ICU studies, diagnostic or stewardship interventions with a reproducible effect on antibiotic use, and ICU-specific consensus recommendations compatible with the primary evidence. A recommendation was not formulated solely from an editorial or opinion source.

Third, each candidate component was assessed for clinical safety, microbiological reliability, applicability to multidrug-resistant Gram-negative infections, dependence on local susceptibility data, staffing requirements, diagnostic availability, and feasibility in a Romanian tertiary-care ICU. The authors reviewed the mapped components, resolved disagreements through discussion, and retained recommendations by consensus. The resulting framework is a preliminary evidence-informed implementation proposal and was not developed using a formal guideline-grading methodology.

We assessed each proposed intervention for clinical safety; microbiological reliability; relevance to MDR Gram-negative epidemiology; diagnostic and resource requirements; staffing and stewardship requirements; feasibility in a Romanian tertiary-care ICU; dependence on local antibiogram data; and the need for infectious-disease, microbiology, pharmacy, or PK/PD expertise.

We excluded Romanian studies lacking eligible ICU de-escalation outcomes from the clinical evidence table, but used them as contextual sources for local antibiotic use, resistance trends, and implementation feasibility. The framework developed was meant to support evidence-based decisions rather than serve as a formal clinical practice guideline. Therefore, local application should include ICU-specific antibiograms, institutional resistance patterns, rapid diagnostic tools, source control strategies, organ dysfunction status, and individual PK/PD factors.

### 4.11. Ethics

As this scoping review used information from previously published studies and did not involve individual patient data, ethics committee approval and individual informed consent were not required.

## 5. Conclusions

De-escalation of antibiotic therapy is not consistently linked to increased mortality rates and may actually reduce the duration of broad-spectrum antibiotic exposure. The predominantly observational evidence, limited randomized data, and substantial potential for confounding and survivor bias preclude conclusions of equivalence, non-inferiority, or survival benefit. However, the lack of a shared operational definition is a significant barrier to synthesizing information and strengthening any recommendations. Additionally, clinicians often underuse de-escalation, even when it is microbiologically appropriate. The ecological benefit frequently cited to support de-escalation—namely, reduced antibiotic resistance—is mostly assumed rather than proven, as resistance emergence is rarely measured. Moreover, class-specific data for antibiotics like colistin and tigecycline remain insufficient to inform prescriptive switching rules. Rather than merely encouraging another attempt at de-escalation, this review presents a concrete, resource-efficient pathway aligned with the WHO’s five-pillar model. This includes an evidence-based framework, a structured 48 to 72 h antibiotic time-out, linking each reassessment to a documented decision, an eligible patient denominator for fair benchmarking, and a minimum monitoring dataset that captures both resistance and ecological outcomes, not just consumption data. This approach was designed for potential application in high-resistance, resource-variable environments, including Romanian tertiary-care ICUs, but its feasibility and effectiveness require prospective evaluation. Successful de-escalation now relies less on additional observational studies and more on standardized definitions, well-designed trials that report resistance and ecological outcomes alongside mortality rates, and the prospective validation of implementation strategies—like those proposed here—in the settings that need them most.

## Figures and Tables

**Table 1:** Characteristics and outcomes of studies on antibiotic de-escalation in critically ill/ICU patients. DE = de-escalation; ADE = antibiotic de-escalation; DOT = days of therapy; LOT = length of therapy; DDD = defined daily dose; LOS = length of stay; NPV/PPV = negative/positive predictive value; NS = not significant; PSM = propensity-score matching; mPCR/FAPP = multiplex PCR/FilmArray pneumonia panel; MDR/XDR = multidrug-/extensively drug-resistant; CRE/CRAB/CRPA = carbapenem-resistant *Enterobacterales*/*A. baumannii*/*P. aeruginosa*; and PCT = procalcitonin, ↓ = decrease; ↑ = increase; → = modifies to.

A. Original comparative studies (RCTs, quasi-experimental & observational cohorts)—*n* = 35						
No.	Author, Year, Region, and Type of Study	Setting, Population, Infection, Microbiology, and Resistance	De-escalation Definition, Strategy, Timing, and Criteria	De-escalation Frequency and Mortality	Clinical, Microbiological, Resistance, and Implementation Outcomes	Authors’ Reported Conclusions
1	Kim 2022—Republic of Korea [19] Retrospective multicenter cohort (end-of-life)	1296 inpatients dying ≤7 d after suspending life-sustaining treatment (2020); 32.9% in ICU. Of 1251 exposed, 213 de-escalated. Pneumonia 23.9%; organisms/resistance not reported.	Narrower agent or stop ≥1 combination component. After formal suspension, before death (median 2 d). No standardized criteria.	DE in 213/1251 (17.0%); complete withdrawal 8.3%. Conversely, 43.6% received broader/more antibiotics. Mortality: 100% by design (not an outcome).	ICU location favored DE (29.6% vs. 10.8%; aRR 2.77, 1.97–3.90). Cure/comfort/eradication/resistance not assessed.	Antimicrobial use stayed excessive in dying patients; antibiotic discontinuation should be an explicit stewardship intervention at end of life.
2	Sekandarzad 2025—Germany [20] Prospective intervention vs. matched retrospective controls (PSM)	400 critically ill with pneumonia (200/200); 60% ventilated, 16% shock, 16% immunosuppressed. CAP 63%, HAP 38%. <72 h stays excluded.	No patient-level definition; at program level ↓ meropenem/pip-tazo, ↑ ampicillin–sulbactam, more targeted therapy. Reconsider empirical Rx within 3 d.	ICU 28-d mortality 28.0% vs. 26.5% (NS); PSM 26.8% vs. 26.5% (*p* = 0.951).	Total DOT 12.95 → 9.91 (−23.6%, *p* = 0.036); meropenem/pip-tazo DOT down, ampicillin-sulbactam up. Respiratory sampling 58% → 79%; pathogen detection 31% → 46%. LOS/ventilation NS. Resistance not evaluated.	A multifaceted ICU stewardship program cut broad-spectrum use in pneumonia without harming mortality, LOS or ventilation; better sampling enabled targeting.
3	Lakbar 2022—France [21] Retrospective observational, historical pre-COVID control	COVID ICU: 170 on antibiotics for suspected superinfection (141 empirical); 87.6% ventilated. Historical: 58 empirical. Predominantly pulmonary; documented in 39%.	Narrower/lower-impact agent or stop a combination component; complete stop not counted as DE. First change between empirical start and micro results; daily rounds.	DE 47/141 (33.3%), escalation 3.5%, continuation 63.1%. ICU/hospital/28-d/90-d mortality NS across strategies.	DE = narrowing 61.7% + stopping components 38.2%. Recurrence 27.7% (DE) vs. 60% (escalation) vs. 9% (continuation), *p* = 0.001. DE lower during COVID than historically (27.6% vs. 52.2%, *p* < 0.001). MDR acquisition NS.	Empirical therapy was mostly appropriate; DE did not worsen mortality/LOS but fell markedly during COVID—workload/organizational stress impaired stewardship.
4	Yu 2023—China [22] Single-center pre–post (AMS), neurosurgical ICU	1013 adults (487 pre/526 during AMS); ~83% post-neurosurgery. MDRO subgroup: 90/58. Respiratory predominant; MDROs: *K. pneumoniae*, *A. baumannii*.	“Appropriate DE” (no exact spectrum definition): empirical → pathogen-/susceptibility-directed. Pharmacist daily; MDD every 3 d; MDRO reviewed 1–2×/d. PCT days 3/7.	Overall mortality 10.46% vs. 12.32% (*p* = 0.373); MDRO subgroup 22.41% vs. 26.67% (NS).	Empirical antipseudomonal β-lactam 60.99% → 43.92% (*p* < 0.001). Appropriate DE overall 22.3% → 28.0% (*p* = 0.072); in MDRO-positive 20% → 39.66% (*p* = 0.001). MDRO infections 18.48% → 11.03%; polymyxin 31% → 15.5%. Gram-negative susceptibility improved.	Pharmacist-led program cut empirical broad-spectrum/polymyxin use, raised appropriate DE in MDRO-positive pts and improved susceptibility without excess mortality.
5	Mishima 2023—Japan [23] Non-randomized before–after with ITS, mixed ICU	2730 admissions (1199 pre/1386 during antibiotic time-out, ATO); survival analysis 777/796. Infection as the primary reason: ~10%. Sources/organisms not reported.	DE not explicitly measured; ATO = structured provider-led reassessment of indication, effect, duration at days 3/7/14 (5 weekdays).	ATO associated with higher survival to hospital discharge (aSHR 1.13, 1.02–1.25, *p* = 0.02); no difference in ICU-discharge survival. Attenuated after DOT adjustment (SHR 0.98).	Total IV DOT fell (intercept −178.26, *p* = 0.02); antipseudomonal ↓ (level + slope); carbapenem ↓ over time; anti-MRSA ↓. Cure/recurrence/resistance not assessed.	Intensivist-driven antibiotic time-outs in ICU rounds cut IV/antipseudomonal/carbapenem/anti-MRSA exposure and improved discharge survival, without special resources.
6	Jover-Sáenz 2020—Spain [24] Prospective before–after with historical control	67,362 antibiotic-treated pts (34,560 pre/32,802 post); ICU sample not separated. Surveillance: MRSA, MDR Acinetobacter, ESBL/carbapenemase *K. pneumoniae*, MDR Pseudomonas.	Empirical broad-spectrum → targeted/narrower per micro + clinical course. Daily review; acceptance at 24–48 h.	Hospital mortality 3.34% vs. 3.14% (*p* = 0.210); ICU-specific not reported.	ICU total antibiotic use 155 → 113 DDD/100 bed-days (*p* = 0.005); carbapenems 21.3 → 9.10, colistin 17.88 → 2.67; 3rd-gen cephalosporins rose. Hospital MDRO incidence and ICU MDR A. baumannii fell.	Long-term stewardship lowered ICU antibiotic consumption, MDRO incidence, and costs without increased mortality.
7	Miller 2023—USA [25] Retrospective quasi-experimental before–after	124 ICU pneumonia pts (66 before/58 after pneumonia-panel). CAP/HAP/VAP; ~50% no pathogen. Deaths/comfort-care excluded.	Narrow/remove empirical components (distinct from full stop) after pneumonia panel (~75 min) + culture reassessment.	DE 71.2% vs. 69.0% (*p* = 0.85). Mortality not evaluated.	Anti-MRSA stop 49.1 vs. 41.8 h (*p* = 0.28); duration 7.9 vs. 7.6 d (NS). In <72 h anti-MRSA subgroup, stop 41.3 vs. 21.3 h (*p* = 0.02). Resistance not evaluated.	The pneumonia panel showed numerical but non-significant DE gains; rapid diagnostics must be paired with education and active stewardship.
8	Ali 2022—Pakistan [26] Prospective interventional, MICU/HDU	134 critically ill on carbapenems with non-adherent prescriptions; 117 accepted vs. 17 rejected ASP advice. Respiratory 41%, sepsis 13%. 48% no culture growth.	ASP-recommended carbapenem modification (not operationally defined). Daily review; day of DE not reported.	Carbapenem DE in 34/134 (25.4%). 30-d mortality 11.1% (accepted) vs. 11.8% (rejected), *p* > 0.999.	7-d clinical improvement 84.6% vs. 70.6% (*p* = 0.171); 30-d readmission lower when accepted (13.7% vs. 35.3%, *p* = 0.036). Physician acceptance 87.3%. Resistance not evaluated.	Carbapenem stewardship was feasible and widely accepted in a resource-limited ICU, with fewer readmissions and no excess mortality.
9	Panditrao 2021—India [27] Prospective before-and-after interventional, surgical semi-ICU	337 adults (94 baseline/243 intervention); complex postoperative + sepsis. Secondary peritonitis 27.6%. Isolates: *A. baumannii*, *K. pneumoniae*, *E. coli*; MDR frequent.	Not standardized; reassess, then narrow/withdraw per clinical + micro. Formal 48 h time-out with repeats.	DE-related recommendations 15.7% → 21.4% (*p* = 0.26). Mortality not reported.	DOT 1112→1049 and LOT 956 → 936/1000 pt-days. Carbapenem use 26.3% → 20.9%; colistin rose (MDR). Double Gram-negative coverage 9.6% → 2.9% (*p* = 0.02). VAP 46.4 → 35.4/1000 vent-days. No longitudinal resistance outcome.	Combining stewardship with time-outs, dose optimization, education and infection control modestly cut exposure and unnecessary double coverage in a resource-limited surgical ICU.
10	Schumann 2021—Germany [28] Before–after observational (rapid diagnostics), ICU/IMC	329 adults, 364 BSI episodes (intervention 179/200; control 150/~166). Sepsis/shock; CoNS, *S. aureus*, Gram-neg, fungi; few ESBL/MRSA.	Not formally defined; broad empirical → narrowest effective/guideline-concordant, or stop if CoNS = contamination. After FilmArray (~70 min).	Mortality NS between groups (*p* = 0.135).	Optimal therapy ~17 h earlier (20 vs. 37 h, *p* = 0.071); any change 24 h earlier (36 vs. 60 h, *p* = 0.029). FilmArray changed 22.2% of episodes. Overall duration was similar; in CoNS, it was unexpectedly longer with FilmArray (166 vs. 117 h, *p* = 0.041). Subsequent resistance not reported.	Rapid BCID enabled earlier adjustment but did not reduce mortality, LOS, or overall antibiotic exposure—rapid results must be linked to active stewardship.
11	Gu 2023—China [29] Retrospective before–after cohort, 1:1 PSM, ICU	561 pre-match (270/291); 102/102 matched (>65 y). Pneumonia 29.9%, sepsis 23.8%, shock 11.2%; organisms/resistance not reported.	Not operationally defined; pharmacist recommendation to “de-escalate in time.” Daily pharmacist review; time to DE not reported.	Mortality 19.6% (control) vs. 22.6% (pharmacist), *p* = 0.607.	833/862 recommendations accepted (96.6%); 75 DE recommendations all accepted. Antibiotic-use density 241.9 → 176.6 DDD/100 bed-days (*p* = 0.018); carbapenem 23.1% → 14.4%. Antibiotic cost $836 → $362/stay (*p* < 0.001). Cure/resistance not reported.	Pharmacist-led stewardship cut consumption and cost without a significant mortality increase; a trained ICU pharmacist improves rational use.
12	Zhu 2025—USA [30] Prospective observational cohort, MICU	686 ventilated pts (927 suspected-pneumonia episodes) undergoing BAL; 30% immunocompromised. CAP/HAP/VAP 150/257/406; 104/486 bacterial episodes resistant. SARS-CoV-2: 76% of viral.	Reduction in breadth/number of antibiotics (declining NAT score); complete stop = NAT −2. PCR ~4 h, susceptibility ≥ 72 h; DE assessed daily to day 7.	DE occurred in all categories except resistant-bacterial by days 1–2. Composite unfavorable outcome (death/hospice/transplant) 43.6%, comparable across aetiologies.	By day 4, ~56–57% of susceptible/microbiology-negative episodes were de-escalated; viral episodes were fully stopped by day 5 (44%). C. difficile: 2%. PCR vs. culture concordant 61.4%.	BAL cultures + rapid multiplex PCR supported prompt de-escalation without increased unfavorable outcomes; protocolized prospective studies needed.
13	Souza-Oliveira 2016—Brazil [31] Retrospective cohort, clinical–surgical ICU	120 VAP pts, 132 episodes; mean age 49. *P. aeruginosa* 30.8%, *S. aureus* 23.8%, *A. baumannii* 19%. MDR 45.6%; carbapenem-R *P. aeruginosa* 47.6%, *A. baumannii* 69.2%.	Stop treatment or replace with a narrower agent after quantitative culture/susceptibility. Daily reassessment advocated.	Maintained 57%, escalated 33%, DE ~10%. Overall mortality 35%; DE 16.7% vs. escalation/maintenance higher, but DE not significantly linked to mortality (*p* = 0.160).	Resistant vs. susceptible mortality NS (27% vs. 46%, *p* = 0.104). Incorrect renal-dose adjustment independently raised mortality (OR 8.76, 1.80–42.53). DE resistance outcomes not reported.	DE was not associated with higher mortality; prescription quality (loading dose and renal adjustment) may matter more than DE or resistance.
14	Aissaoui 2025—Morocco [32] Prospective multicenter observational, 12 ICUs	210 ventilated pneumonia pts; median APACHE II 15; shock 35%. CAP 30%, VAP 58%, HAP 12%. mPCR: 88 resistance genes (CTX-M 32, NDM 26, OXA-48 10).	Narrowing/lower-impact or stopping combination components; cessation analyzed with DE. mPCR turnaround 2 h.	Treatment modified in 58%: DE/cessation 11%, escalation 26.5%, initiation 13%. ICU mortality ~51%; appropriate post-mPCR therapy independently lowered mortality (aOR 0.37, 0.15–0.93).	mPCR sensitivity 96.9%, NPV 99.9%. Appropriate treatment rose 38.7%→67% (*p* < 0.0001). Experts flagged an extra 26% who could have been de-escalated. Resistance not evaluated.	mPCR improved empirical-therapy appropriateness and supported de-escalation, but missed opportunities remained; rapid diagnostics need stewardship expertise.
15	Roper 2023—USA [33] Retrospective cohort (culture-negative), multiple ICUs	173 adults on broad-spectrum ≥ 5 d despite negative cultures; 53% ventilated. Presumed pneumonia 51%, sepsis of unknown source 26%. Resistance unavailable.	Spectrum narrowing ≤72 h: pivotal (e.g., meropenem → cefepime) + companion (drop MRSA/double antipseudomonal).	Pivotal DE 38/173 (22.0%); companion drop 47.4% (98% anti-MRSA). ICU mortality 7.9% vs. 15.6% (*p* = 0.227); inpatient 15.8% vs. 19.3% (NS).	DE → shorter pivotal (3 vs. 6 d) and total therapy (6 vs. 8 d, *p* = 0.003); hospital LOS 9 vs. 17 d and ICU LOS 5 vs. 9 d (*p* < 0.001); lower AKI (10.5% vs. 27.4%, *p* = 0.031). No difference in escalation/HAI/CDI/resistance.	Early pivotal + companion DE was feasible in culture-negative critically ill pts without increased mortality/failure, and shortened therapy/LOS with less AKI.
16	Rodríguez-Gómez 2025—Spain [34] Prospective observational cohort, mixed ICU	236 adults, 344 suspected nosocomial LRTI episodes; APACHE II 20.9; MDR risk in 86%. HAP 49%, VAP 20%, VAT 31%. Genes: VIM 11, KPC 4, CTX-M 3.	Stop an agent or narrow spectrum after pneumonia-panel result (~1 h).	PCR influenced prescribing in 57.6% of episodes; of 255 already on antibiotics, 60.8% modified (69.6% PCR-concordant). ICU mortality 28.8%, not compared by DE.	PCR sensitivity 93.4%, NPV 97.9%. Exact DE/discontinuation numbers not separated. Duration, cure, recurrence, resistance not evaluated.	Multiplex pneumonia PCR strongly influenced real-world prescribing, most useful for rapidly excluding bacterial infection; interpret with culture and context.
17	Aldardeer 2023—Saudi Arabia [35] Two-center retrospective comparative cohort, ICU	250 adults on empirical antipseudomonal ≥ 48 h; 125 DE/125 continuation; 58% ventilated. Cultures positive 39.6%; ESBL 24, CRE 14.	Replace broad-spectrum with narrower agent; dropping one antipseudomonal or MRSA cover alone not counted. Reassess within 72 h/24 h of cultures.	Only 25.6% de-escalated within 48–72 h. ICU mortality 33.6% vs. 40.0% (*p* = 0.294); in-hospital 39.2% vs. 45.6% (NS).	Superinfection 6.4% vs. 10.4% (*p* = 0.254). Broad-spectrum duration was shorter with DE (7.2 vs. 10.3 d, *p* < 0.001), but total duration was similar. Hospital/ICU LOS was longer with DE. Re-escalation in 57/125. Acquired resistance NS.	DE was not associated with different superinfection/mortality vs. continuation; narrowing was generally safe. Earlier DE with rapid diagnostics should be tested in high-resistance ICUs.
18	Le 2024—Vietnam [36] Retrospective observational (culture-negative VAP)	43 ventilated adults, suspected VAP, negative quantitative ETA cultures, early stop; median age 76; APACHE II 18.	Complete withdrawal of all antibiotics within 24 h of final negative culture (cessation, not narrowing). ~3 d empirical therapy.	Overall mortality 18.6%; 16.1% after successful stop vs. 25.0% with failure (*p* = 0.665).	Treatment failure/recurrent VAP 12/43 (27.9%) within 48 h. Higher mCPIS predicted failure (OR 1.66); mCPIS + PCT OR 1.77 (AUC 0.765); PCT alone was poor. Resistance not evaluated.	Early stop in culture-negative VAP carried a substantial failure rate; PCT alone should not guide cessation—combine mCPIS with PCT.
19	Arulappen 2025—Malaysia [37] Single-center 5-year retrospective cohort, MICU	1134 adults on empirical broad-spectrum then de-escalated; 55% severe sepsis, 45% shock; 47% vasopressors. Pulmonary: 80%; cultures negative at 72 h in 82.9%.	Replace empirical broad-spectrum with narrower agent (culture-directed changes excluded). ~3 d before modification (WHO LMIC protocol).	105/1134 died (9.3%). Independent mortality: HAI (aOR 12.56), SOFA ≥ 6 (aOR 21.44), ≥1 vasopressor (aOR 38.46); higher SBP protective.	Adequate source control 86.2%; infection-free 87.7%; 30-d readmission 3.4%. Resistance/CDI not evaluated.	Empirical DE should be encouraged but individualized by HAI, hemodynamics, SOFA, and vasopressor needs; identified factors could seed a DE decision tool.
20	Trupka 2017—USA [38] Prospective cross-over interventional, 2 MICUs	283 ventilated adults, suspected pneumonia; APACHE II ~22. Enhanced DE team (EAD, 144) vs. routine (RAM, 139). Pathogen-negative 35%, viral 17%.	Reduce number of antibiotics/drop a pathogen category/narrow regimen; Gram-negative ranked carbapenem → ceftriaxone. Daily weekday review; early failures excluded.	Among eligible, DE 67.3% (EAD) vs. 66.0% (RAM), *p* = 0.845. Hospital mortality 35.4% vs. 25.2% (*p* = 0.061). By decision: DE 16.4% vs. no change 43.3% vs. escalation 50% (confounded).	Total duration: 7 d in both. Post-DE deterioration 11.4% vs. 8.6% (NS); resistant secondary pneumonia 6.3% vs. 4.3% (NS). DE more frequent without shock (61.6% vs. 44.6%, *p* = 0.001).	Adding an enhanced DE team to an ICU already practicing strong stewardship did not increase DE or shorten therapy (ceiling effect); daily review remains routine.
21	Ghosh 2023—India [39] Retrospective secondary analysis of prospective database (ANT-CRITIC)	276 empirical-prescription episodes, ICU ≥ 72 h/until cultures. Culture-positive DE in 41. Pulmonary 50.7%, urinary 22.8%. Organisms not reported.	In culture-positive: drop a non-pivotal agent and/or narrow the pivotal agent (spectrum-scoring system). Reassess at/after 72 h.	Overall mortality 34.1%. Culture-positive DE 24.4% vs. culture-positive/no-DE 48.7%. Positive culture without DE independently predicted mortality (aOR 2.77, 1.18–6.53).	Day-7 cure in DE group: 78.1%; SOFA change: +0.76. Therapy longer after DE (8.27 ± 4.11 d). Resistance/superinfection not evaluated.	Failure to de-escalate culture-positive pts on appropriate therapy independently predicted mortality (reflecting resistance/instability); DE linked to better outcomes but longer courses.
22	Choudhuri 2021—India [40] Retrospective observational cohort, mixed ICU	76 critically ill de-escalated: before day 8 (41) vs. day 8+ (35). Mixed infections. New resistant organisms: ESBL, CR-Pseudomonas/Acinetobacter, MRSA, VRE.	Stop broad-spectrum or narrow after clinical improvement/PCT/cultures. “Normal” DE before day 8; late = day 8+. Exposure 6 ± 1.5 vs. 11 ± 3.4 d (*p* = 0.04).	ICU mortality 7.3% (early) vs. 11.4% (late), *p* = 0.14. Prior 30-day antibiotic exposure predicted mortality in the late group (OR 2.34).	New infections 19.5% vs. 34.2% (*p* = 0.04); new resistant organisms 9.7% vs. 25.8% (*p* = 0.02); ICU LOS 11 vs. 14 d (*p* = 0.06).	Late DE (driven by persistent infection) brought more new infections/resistant organisms, though not higher mortality; recent antibiotic exposure raised mortality risk.
23	Contier 2025—France [41] Retrospective cohort (immunocompromised), ICU	114 immunocompromised adults, hypoxaemic ARF, ventilated, suspected pneumonia; SAPS II 53, SOFA 8.5. Early sampling (VAP excluded). Enterobacterales predominant; 1 ESBL, no MRSA/carbapenemase.	Stop one antibiotic or narrow ongoing therapy (dropping anti-staph counted as DE). PCR ~2.5–4 h vs. 48–72 h culture.	PCR changed therapy in 20/114 (17.5%): DE 11, cessation 3, escalation 2, initiation 2. ICU mortality 39.5%; not reported by DE status.	Modifications appropriate retrospectively in 89.5%. Conventional cultures changed therapy in 25 (15 DE). Pneumonia resolved in 62.3%. PCR NPV 98%; carbapenems most spared.	Multiplex PCR (excellent NPV) enabled early modification, mainly DE, in ~1/5 of immunocompromised ICU pts, but must complement culture.
24	De Bus 2020 (DIANA)—28 countries, incl. Romania [18] Prospective international multicenter observational, 152 ICUs	1495 adults on empirical therapy for suspected/confirmed infection. ADE ≤ 3 d in 240 (16.1%), no change 62.5%. Respiratory 48%, abdominal 18%; 55.8% micro-confirmed; baseline MDR colonization 11.5%.	Within 3 d: drop unnecessary combination components or replace an agent intending to narrow spectrum. ADE spread across days 0–3.	ADE 16.1%. 28-d mortality 15.8% (ADE) vs. 19.4% (no change), RR 0.83 (0.60–1.14), *p* = 0.27; ICU mortality 11.7% vs. 15.5% (NS).	Day-7 cure 57.9% vs. 42.7%, RR 1.34 (1.18–1.52); IPW RR 1.37. MDR emergence 7.5% vs. 11.9%, RR 0.63 (*p* = 0.06). Antimicrobial-free days/duration similar. Only 25.4% of ICUs had local ADE guidelines.	ADE applied in only 16% within 3 d; adjusted estimates showed no reduction in cure (possible benefit), but causality uncertain (residual confounding). [Flagship cohort].
25	Latorre Ibars 2026—Spain [42] Two-center retrospective cohort (rapid PCR), ICU	363 respiratory samples from 261 critically ill patients; 88% during ventilation; 50% suspected nosocomial. FAPP: H. influenzae 18.5%, S. aureus 12.1%. Panel included CTX-M/KPC/NDM/OXA-48.	FAPP-prompted “negative” change = narrow spectrum or stop ≥1 antibiotic.	Therapy changed 108/363 (29.8%): DE/discontinuation 75 (20.7% of samples), initiation/escalation 33. Mortality not reported.	FAPP positivity 65.3% vs. culture 23.1%; sensitivity 98.8%, NPV 99.2%; agreement κ = 0.27. Among FAPP-neg/culture-neg, 26.6% narrowed/stopped. LOS/duration/resistance not assessed.	FAPP rapidly influenced decisions in ~1/3 of ICU cases, mainly DE/discontinuation; clinical judgment and cultures remain essential.
26	Kuwana 2020—Japan [43] Retrospective sequential case series (ESBL bacteremia)	25 ICU sepsis pts, ESBL-Enterobacterales bacteraemia; 15/25 shock; 11 de-escalated to cefmetazole, 14 continued. Source UTI 56%; *E. coli* 85%.	Replace empirical broad-spectrum with narrower active agent (cefmetazole). Median day 4 (range 3–6).	All 11 cefmetazole-DE pts survived vs. 8/14 without DE; all 6 deaths in the non-DE group. UTI mortality 0% in both.	No recurrent shock/failure after DE; all DE isolates were cefmetazole-susceptible. Best outcomes in urinary-source *E. coli*. No adjusted comparison.	Cefmetazole may be a carbapenem-sparing definitive option for ESBL–*Enterobacterales* bacteremia (esp. urinary-source *E. coli*) once stable and susceptibility confirmed.
27	Kwon 2019—South Korea [44] Retrospective cohort (culture-negative), MICU	107 adults, culture-negative severe pneumonia + sepsis/shock; 40 DE/67 no-DE; 91% ventilated; APACHE II 20, SOFA 9.6. HAP 97/107.	Drop a pivotal/companion antibiotic, or replace the carbapenem/antipseudomonal agent, by ICU day 5. Median 3 d; all by day 5. No formal protocol.	ICU mortality 27.5% vs. 41.8% (*p* = 0.137); aHR 0.739 (0.317–1.723). In-hospital 37.5% vs. 55.2% (*p* = 0.076).	LOS 11.5 vs. 10 d (NS); duration 21 vs. 24 d (NS); antibiotic burden lower with DE (11.0 vs. 12.6, *p* = 0.050). MDR occurrence 15% vs. 16.9% (NS); CDI 7.5% in both.	DE in culture-negative pneumonia with sepsis/shock was not linked to higher mortality or MDR and reduced antibiotic burden—consider it when cultures stay negative through day 5.
28	Yoon 2026—South Korea [45] Retrospective observational (rapid PCR), MICU	75 adults, HAP/VAP with mPCR + culture; 97% ventilated; APACHE II 25, SOFA 10. mPCR detected bacteria in 34; genes: CTX-M/KPC/NDM. Prior CRE/CPE colonization: 18.7%.	Stop carbapenem or anti-MRSA per mPCR; switching to ceftazidime–avibactam/ceftolozane–tazobactam not counted. mPCR modification 5.8 h vs. 122.3 h (*p* < 0.01).	In-hospital mortality 62.7%; mPCR-guided modification not independently linked to mortality; DE outcomes not separately reported.	Among 24 carbapenem-treated with negative Gram-negative mPCR, stopped in only 1 (4.2%); among 39 on vanco/teico with negative S. aureus mPCR, stopped in 3 (7.7%). mPCR was not linked to resistant-organism acquisition.	mPCR shortened ID/modification time but carbapenem/anti-MRSA DE stayed rare—molecular testing alone is insufficient; structured stewardship is required.
29	Ferrer 2018—Spain [46] Prospective multicenter before–after QI, 72 ICUs	2628 adults, severe sepsis/septic shock; APACHE II 22, SOFA ~8.6. Pneumonia 857, abdominal 883, urinary 438. Community → ICU-acquired.	Switch/stop an antibiotic class to a less broad-spectrum agent after cultures. Formal reassessment at ~72 h.	DE rose 16.3% → 20.1% (*p* = 0.004). Hospital mortality 30.5% → 29.4% (*p* = 0.544); intervention not associated with mortality (aOR 1.08).	Inappropriate empirical therapy fell from 8.9% to 6.5%; time to antibiotics fell from 2.5 to 2.0 h. LOS unchanged. DE gains sustained at follow-up. Resistance/cure not evaluated.	A multifaceted educational intervention improved timeliness/appropriateness and increased DE (sustained), but did not reduce mortality.
30	Salahuddin 2016—Saudi Arabia [47] Prospective single-center observational	395 critically ill with sepsis/septic shock; APACHE II 24. 195 culture-positive (BSI 42%, respiratory 37%); MDR in 41/195; 200 culture-negative. DNR/imminent death excluded.	Stop an agent or change to a narrower spectrum; strategy classified on ICU day 7.	DE in 189/395 (48%); no change 39%, escalation 11%. ICU mortality 18.7%; 4-group 14.8% (DE) vs. 24.4% (no change), *p* = 0.11 (unadjusted).	Empirical therapy appropriate in 57%. ICU LOS differed across groups (*p* = 0.003). Resistance/recurrence/duration not evaluated.	DE performed in <half; clinicians reluctant with greater severity, hematological malignancy, fungal sepsis, or MDR. Formal ASP could raise DE confidence.
31	Li 2018—China [48] Retrospective cohort, 1:1 PSM (trauma VAP)	156 ventilated trauma-VAP pts; 62 DE/94 no-DE; 42/42 matched. Organisms *P. aeruginosa*, *A. baumannii*, *K. pneumoniae*; ESBL, MRSA, MDR.	Broad-spectrum → narrower per culture (combination → monotherapy, narrowing or stop). After micro results; exact day not reported.	After matching, 28-d mortality 28.6% (DE) vs. 23.8% (no-DE), *p* = 0.620; DE not independently linked to mortality (OR 0.51, 0.25–1.03).	Duration shorter with DE (11 vs. 14 d, *p* = 0.045); antibiotic cost 6430 vs. 7618 RMB (*p* = 0.043); hospital cost lower. LOS/ventilation NS. Post-treatment MDR 31% vs. 40.5% (NS).	In trauma-VAP, DE cut duration and costs without increasing mortality, LOS, ventilation, or MDR; RCTs needed to confirm.
32	Niimura 2018—Japan [49] Retrospective observational cohort, EICU	85 ambulance-transported sepsis pts; DE appropriate in 60, performed in 21 (35%). 41 blood-culture-positive; 30 septic shock.	Narrower agent or fewer antibiotics after pathogen ID/susceptibility. Median organism ID day 4 (DE) vs. 3 (no-DE).	DE 21/60 eligible. Mortality 9.5% vs. 23.1% (*p* = 0.227); septic shock 10% vs. 40% (*p* = 0.204); culture-positive 12.5% vs. 24% (*p* = 0.448).	Shorter stay with DE (12 vs. 26 d, *p* = 0.028), pronounced in culture-positive (11 vs. 26 d, *p* = 0.030); duration 12 vs. 16 d (*p* = 0.071). Resistance not evaluated.	DE was associated with shorter hospitalization/exposure (esp. culture-positive) without excess mortality; culture-guided narrowing may improve efficiency.
33	Rodrigues 2019—Brazil [50] RCT (superiority), cardiac tertiary ICU	200 adults, hospital-acquired sepsis ≥ 48 h; ~89% severe sepsis/shock. SeptiFast-guided (100) vs. blood-culture-guided (100). BSI, pneumonia/VAP, other. Prior MDR colonization: 30%.	Switch to a narrower agent or reduce to monotherapy. SeptiFast result 6–12 h; median adjustment 8 h vs. 54 h with culture.	DE 89.5% vs. 84.0% (*p* = 0.700), but earlier with SeptiFast. Mortality NS: 28-d 40% vs. 47% (*p* = 0.318); hospital 55% vs. 61%.	Overall DOT NS (1621 vs. 2000, *p* = 0.067); in culture-positive, lower with SeptiFast (1429 vs. 1889, *p* = 0.017) and shorter therapy (12 vs. 15 d, *p* = 0.039). Re-escalation 10.5% vs. 28.0%. Resistance not evaluated.	SeptiFast did not cut overall consumption, but positive rapid results enabled earlier de-escalation and shorter therapy without worse outcomes.
34	Sellers 2021—USA [51] Retrospective diagnostic cohort, MICU	1300 MICU pts; 1567 blood + 514 respiratory + 1059 urine cultures; 85.5% immunocompetent. True infection in 58.3% of blood, 88.9% of respiratory, 84.2% of urine.	No treatment definition; DE-eligible at 72 h if cultures negative + clinical-stability criteria (normothermia, WBC 4–12, decreasing vasopressor).	Only 17% met all clinical DE criteria. Descriptive hospital mortality 17.6–23.7%; not compared by DE.	NPV of negative 72-h culture: 0.99 blood, 0.82 respiratory, 0.97 urine. Clinical criteria predicted finalized-negative cultures with sensitivity 0.88, specificity 1.00. Actual changes/cure/resistance not evaluated.	Negative blood/urine cultures at 72 h + clinical stability support protocolized reassessment; read negative respiratory cultures cautiously in suspected pneumonia.
35	Nikolai 2026—Germany [52] Secondary analysis of two prospective multicenter cohorts (ICU subgroup)	ICU subgroup: 145 clinically stable adults, monomicrobial BSI, narrower option available at day 5; 103 DE/42 no-DE. MDR: 7.1%. Various sources.	Empirical combination → monotherapy or narrower agent by day 5 (WHO AWaRe hierarchy). Assessed on day 5.	ICU DE rate 71.0% (103/145). ICU-specific mortality not reported; whole cohort in-hospital 11.3% vs. 9.3% (*p* = 0.369).	ICU treatment favored DE (aOR for non-DE 0.60, 0.38–0.95). Whole cohort: unnecessary carbapenem continuation and low DE for urogenital/E. were concerns. ICU cure/duration/resistance not separately reported.	DE opportunities frequently missed despite stability/available susceptibility; ICU pts de-escalated more often—target unnecessary carbapenem continuation and Gram-neg BSI.
B. Systematic reviews & meta-analyses—*n* = 2						
No.	Author, Year, Region, and Type of Study	Setting, Population, Infection, Microbiology, and Resistance	De-escalation Definition, Strategy, Timing, and Criteria	De-escalation Frequency and Mortality	Clinical, Microbiological, Resistance, and Implementation Outcomes	Authors’ Reported Conclusions
36	Lakbar 2020—multinational [4] Systematic review (PROSPERO CRD42020169433)	1 RCT (~120) + 20 observational ICU studies (~84–2658 pts); medical/surgical/mixed ICUs. Sepsis, VAP, BSI, intra-abdominal; susceptible + MDR.	Replace broad-spectrum with narrower/lower-ecological-impact agent or stop combination components; complete stop when infection is excluded and not counted. Daily reassessment ~day 3.	RCT 28-day mortality 31% (DE) vs. 23% (continuation), *p* = 0.55. 14 observational NS, 6 favored DE, none showed harm. Unadjusted synthesis RR 0.71 (0.63–0.80).	RCT: longer therapy + more superinfection (27% vs. 11%, *p* = 0.03); LOS MDR emergence NS across cohorts. Evidence low quality.	DE appears clinically safe, but evidence is low quality; do it early when >5–7 d therapy expected; effects on resistance/microbiota remain unclear.
37	Ohji 2016—multinational [53] Systematic review + random-effects meta-analysis (PRISMA/MOOSE, GRADE)	23 comparative studies; ICU evidence: HAP RCT (109), VAP cohorts (up to 879 pooled), sepsis/shock RCT (116) + PSM cohort (465), 101 neutropenic. Populations overlap.	Appropriate broad-spectrum → narrower by replacement and/or dropping unnecessary components per culture. Timing varied; no uniform reassessment imposed.	ICU-associated pneumonia OR 0.34 (0.17–0.68, low quality). VAP in-hospital OR 0.88 (0.54–1.42). Sepsis/shock RCT HR 1.31 (0.64–2.67). Neutropenic HR 0.51 (0.20–1.33).	DE generally did not worsen mortality; no consistent effect on LOS. One HAP RCT reported more post-treatment MDR and higher MRSA-pneumonia mortality with DE; others did not. Cure/duration/resistance not pooled.	DE appeared safe and potentially effective for several ICU infections, but mortality-benefit evidence was inconsistent—higher-quality studies needed.
C. Narrative reviews, expert viewpoints, editorials & consensus statements—*n* = 14						
No.	Author, Year, Region, and Type of Study	Setting, Population, Infection, Microbiology, and Resistance	De-escalation Definition, Strategy, Timing, and Criteria	De-escalation Frequency and Mortality	Clinical, Microbiological, Resistance, and Implementation Outcomes	Authors’ Reported Conclusions
38	Gardner 2025—multiple countries [54] Narrative review (15 primary studies; 80% observational)	15 studies/5283 pts on carbapenems; 10 studies/542 critically ill (~10%). UTI, pneumonia, intra-abdominal, BSI; Gram-negative (Enterobacterales, Pseudomonas, Acinetobacter); ESBL/AmpC/CRE/CRAB.	Narrow or stop a carbapenem used empirically/definitively (excl. group 2 → ertapenem). Timing varied (days to weeks); stewardship review often 48–72 h.	All 15 reported mortality—mostly similar between DE and continuation (0–35%); 3 showed ~15–22% reductions (likely selection bias). No consistent mortality safety signal in critically ill.	Carbapenem exposure cut ~2–5 d; clinical success mostly unchanged (77–89%); LOS similar/shorter. CDI NS in 4 studies; resistance mostly no difference (1 fewer CRAB after DE).	Carbapenem DE reduces exposure without consistently increasing failure/mortality; individualize by stability, source, immune status, prior exposure, and micro. Evidence in critically ill is limited.
39	Ture 2023—Turkey [55] Narrative review	No pooled sample; adult ICU examples (DURAPOP 410; PCT ICU trial 1575). Lung/intra-abdominal/urinary/BSI/sepsis; MDR Gram-neg (ESBL/CRE, MDR Pseudomonas/Acinetobacter).	Narrow/stop empirical broad-spectrum per daily assessment + micro; shortening and biomarker-guided stopping included. Reassessment 48–72 h.	Surgical-ICU evidence and short-course intra-abdominal treatment showed no mortality increase. PCT-guided care: no significant hospital-mortality difference.	Reduced exposure/duration (PCT cut duration 7 → 5 d in one trial). Cure and LOS generally unchanged. Some resistance improvements not attributable to DE specifically.	Review empirical broad-spectrum daily and narrow/stop when clinical + micro permit; shorter courses and biomarker-supported stopping reduce exposure without worsening major outcomes.
40	Giamarellou 2023—Greece [56] Narrative review (aggregated prospective ICU studies)	*n* = 1757: Routsi (262 sepsis/shock, documented infection) + DIANA (1495). High resistance: resistant pathogens in 62.9% (49% XDR).	Stop ≥1 empirical agent, reduce number, or narrow within 3–5 d of empirical therapy. Routsi ≤ 5 d; DIANA ≤ 3 d.	Routsi (PSM): 28-d mortality 13.3% (DE) vs. 36.7% (no DE), *p* = 0.006. DIANA: no harmful mortality effect (no numbers given).	Routsi: DE feasible in 165/262 but done in only 22.9%. DIANA: 16% de-escalated; cure not harmed. Comparative eradication/recurrence/resistance not reported.	DE is essential ICU stewardship (narrow within 3–5 d) and appears safe, but remains underused—even in high-MDR ICUs.
41	Di Bella 2020—Italy/Slovenia [57] Narrative, practice-oriented review (13 AMS scenarios)	Illustrative ICU case (septic shock, *S. pyogenes*) + PCT RCT (1546 critically ill; 761 PCT-guided/785 standard).	Replace broad-spectrum with a narrower active agent after ID; PCT-guided stopping as an additional strategy (PCT ↓ ≥ 80% from peak or ≤0.5 μg/L).	PCT RCT (per-protocol): mortality 20% (PCT) vs. 27% (standard), *p* = 0.0154.	Median duration 5 d (PCT) vs. 7 d (standard), *p* < 0.0001. Eradication/recurrence/resistance not reported.	Spectrum narrowing is a central AMS intervention driven by ID + local resistance; PCT can support earlier stopping and cut exposure without evidence of harm.
42	Tanzarella 2024—Italy/Spain [17] Narrative review of ICU DE evidence	Multiple ICU studies incl. SR of 14/2461, DIANA (1495), RCT (116), IAI cohort (311), β-lactam study (478). Sepsis/shock, VAP/HAP, BSI, intra-abdominal, fungal, neutropenic.	Replace broad-spectrum with narrower/lower-impact agent or stop unnecessary components; complete stop when infection is excluded and not “classical” DE. Daily; usually within first 3 d.	Meta-analysis mortality RR 0.68 (0.52–0.88). DIANA 28-d 15.8% vs. 19.4% (*p* = 0.27). IAI HR 0.57 (0.25–1.28).	DIANA DE 16%; cure not harmed. RCT: longer therapy + more superinfections, no LOS difference. Resistance after β-lactam DE 30.6% vs. 23.5% (NS); DIANA MDR emergence 7.5% vs. 11.9% (*p* = 0.052).	DE appears safe when supported by appropriate initial therapy, reliable sampling, and reassessment; daily evaluation recommended.
43	De Waele 2020—Belgium/NL/SI/FR [58] Expert viewpoint + narrative review	No cohort; ICU observational studies, trials, expert consensus. Severe sepsis, heterogeneous ICU infections; MDR Gram-neg (ESBL, CR-Acinetobacter).	Stop ≥1 empirical combination component and/or replace broad-spectrum with narrower agent. Generally 48–72 h with susceptibilities; daily reassessment.	Randomized evidence reviewed: similar mortality with DE vs. continuation (exact rates not given).	DE cuts broad-spectrum exposure but sometimes requires longer total treatment. No convincing MDR-emergence reduction (one study: small CR-Acinetobacter colonization reduction).	DE is a core ICU stewardship strategy; its safety is reasonable, but effects on resistance are unproven—it should not justify overly broad empirical therapy.
44	Shirazi 2020—Malaysia [59] Narrative review of inpatient stewardship	48 inpatient stewardship studies; ICU sample not separated. VAP + MDR Acinetobacter/Gram-neg in ventilated ICU pts.	Stop unnecessary broad-spectrum agents or replace with narrower culture-directed therapy. During audit/after micro; some ~48 h.	In the reviewed ICU VAP DE study, mortality differences were NS (no numbers).	ICU VAP DE did not change LOS. ICU prospective audit reduced antibiotic use; carbapenem restriction cut consumption and improved MDR A. baumannii control (no numbers).	Inpatient stewardship improves use, cost, and resistance; hospitals should adapt coordinated multidisciplinary interventions to their needs.
45	Seok 2020—South Korea [60] Narrative review (sepsis therapy/stewardship)	No cohort; ICU RCTs, observational studies, SRs, guidelines. Sepsis/shock, pneumonia/VAP, urinary, BSI, intra-abdominal; culture-positive/negative; MDR.	Replace broad-spectrum with narrower/lower-impact agent, convert double coverage to monotherapy, or stop empirical coverage for unisolated pathogens. Daily; usually 48–72 h.	RCT evidence: no significant mortality difference DE vs. continuation; meta-analyses suggested lower mortality (attributed partly to selection bias).	Broad-spectrum use fell with DE. One RCT: longer ICU stay (15.2 vs. 11.8 d) and therapy (14.1 vs. 9.9 d). No significant MDR-acquisition reduction.	Start prompt, appropriate broad-spectrum therapy in shock, then reassess and de-escalate/stop when micro + clinical findings allow; multidisciplinary ICU stewardship is essential.
46	Moniz 2021—Portugal/Denmark [61] Narrative review (stewardship, biomarkers, PK/PD)	Critically ill adults; DIANA (1495), Leone RCT (116), PCT trials (PRORATA 621, SAPS 1575), CRP RCT (130). Sepsis, VAP/VAT, CAP/HAP.	Stop unnecessary combination components or narrow per improvement + micro. Daily, ideally within 24 h of susceptibilities; biomarker stopping days 3–7.	DIANA: no effect on 28-day mortality. PCT trials NS (PRORATA 21% vs. 20%; SAPS 20% vs. 25%, *p* = 0.0122). Overall benefit uncertain.	DIANA higher day-7 cure with ADE, no LOS benefit. Leone: longer therapy + more superinfections after ADE. PCT cut duration; CRP raised, stopping by day 5. ADE effect on resistance undetermined.	ADE is probably safe but should be evaluated separately from duration; CRP/PCT support stopping with clinical/micro context; PK/PD + TDM reduce inappropriate use.
47	Mokrani 2023—France [62] Structured narrative review (systematic Medline searches)	ICU studies incl. cohort of 1109 (397 carbapenem-DE-eligible), RCT (118), cohort (615), VAP cohort (182). Sepsis/shock, VAP/HAP, BSI; ESBL/AmpC/CRE.	Stop when infection is excluded; switch from combination to monotherapy, narrow spectrum, or replace carbapenems/newer β-lactams with targeted agents. Daily; monotherapy/narrowing usually days 2–3.	Observational studies: no mortality increase with DE. 118 pt RCT: similar outcomes. MERINO (mostly non-ICU): 12.3% pip-tazo vs. 3.7% meropenem (methodological concerns).	Carbapenem narrowing in only 14.9% of eligible patients. RCT: longer duration (9 vs. 7.5 d) but less antipseudomonal/combination. Resistance similar; monotherapy ~ combination.	Narrowing to the most targeted active agent is generally safe/feasible once micro available; combination → monotherapy by days 2–3; carbapenem-sparing for ESBL individualized.
48	Matuszak 2025—USA [63] Narrative review (ICU DE + implementation)	Critically ill; DIANA (1495), opt-out discontinuation trial (10 US hospitals), PRORATA, ventilated cohorts, rapid-diagnostic studies. Broad infection spectrum incl. culture-negative.	Replace broad-spectrum with narrower/lower-impact agent or stop unnecessary combination/empirical coverage. Daily; DE within 24 h of definitive susceptibilities; rapid diagnostics earlier.	DIANA: no 28-day mortality difference. Rapid-diagnostic/MRSA-screening studies: no mortality increase.	DIANA day-7 cure higher (57.9% vs. 42.7%). Opt-out: 32% lower odds of continuing antibiotics. Pneumonia PCR cut inappropriate duration by 45%. β-lactam DE: lowest new Gram-negative resistance (1.42, 1.16–1.68). Stewardship incl. DE cut CDI 32%.	DE appears safe in critically ill and should be assessed continually—within 24 h of susceptibilities (earlier with reliable rapid diagnostics) plus shortest effective duration.
49	Micek 2025—USA [64] Narrative review (MEDLINE, ICU optimization)	ICU studies; DIANA (1495), small RCTs, cohorts, SRs, rapid-diagnostic studies. Sepsis/shock, CAP, HAP/VAP, BSI, culture-negative; MDR incl. MRSA/VRE/CRE.	Replace broad-spectrum agents with narrower options, drop unnecessary combination components, or stop antibiotics if infection is unlikely. Daily; DE within 24 h of susceptibilities; rapid mPCR ~5 h.	DIANA: no significant day-7/28 mortality difference. A SR reported lower mortality with DE (RR 0.68, 0.52–0.88). No original pooled analysis.	DIANA: 16% DE by day 3, higher cure, no mortality disadvantage. Rapid diagnostics NPV ~92–>99%. Culture-negative studies support withdrawing broad-spectrum/anti-MRSA. Resistance outcomes heterogeneous.	Combine timely appropriate empirical therapy, PK/PD, micro testing, prompt DE, short courses and multidisciplinary stewardship; assess and implement DE when appropriate.
50	Benoit 2016—Belgium/Australia/France [65] Focus editorial (ICU adequacy + DE)	3 principal studies: Garnacho–Montero (628 severe sepsis/shock; DE 34.9%), Leone RCT (116), Mokart (101 neutropenic; DE 44%).	Narrow the empirical regimen to the identified pathogen + susceptibility after micro results and clinical-response assessment. Exact reassessment times inconsistent.	Garnacho–Montero: DE linked to lower in-hospital/90-d mortality. Leone: DE did not worsen mortality. Mokart: no excess mortality after adjustment (exact rates not given).	Leone: no worse outcomes but more superinfections after DE. Mokart: 44% DE in neutropenic sepsis without adverse effects. No pooled estimates of cure/LOS/resistance.	DE based on susceptibility should be strongly encouraged after adequate empirical therapy; appears safe (incl. neutropenic), but large multicenter RCTs are required.
51	Cortegiani 2023—Italy [66] Multidisciplinary expert consensus (modified nominal group; SIAARTI)	No cohort; 10 experts (ICU, ID, pharmacology, microbiology). Sepsis/shock, VAP/HAP, severe CAP, BSI, complicated intra-abdominal; MDR incl. KPC/NDM *K. pneumoniae*.	No formal definition; broad empirical → “quasi-targeted”/pathogen-directed after rapid ID + resistance characterization, avoiding unnecessary spectrum/duration. Continuous reassessment; rapid ID ~4 h, susceptibility ~8 h.	No original/pooled mortality analysis; cites the literature supporting prompt appropriate therapy.	No original outcomes. Panel considered rapid diagnostics able to shorten empirical duration and individualized short courses feasible for selected BSI/respiratory/intra-abdominal infections; shorter therapy may reduce MDR selection.	ICU stewardship should combine rapid pathogen-directed therapy, individualized duration, surveillance, PK/PD dosing and multidisciplinary decisions—not mere dose/cost reduction.

**Table 2:** Definitions of de-escalation.

Component of De-Escalation	Application in the Included Literature
Spectrum narrowing	Replacement of empirical broad-spectrum therapy with a narrower active agent
Combination reduction	Discontinuation of one or more components of combination therapy
Complete discontinuation	Included in some studies when infection was excluded; analyzed separately or excluded from the conventional definition in others
Spectrum-ranking approach	Movement from a higher-spectrum or higher-ecological-impact agent to a lower-ranked agent
Carbapenem-specific de-escalation	Discontinuation of a carbapenem or replacement with a susceptible carbapenem-sparing agent
Intravenous-to-oral switch	Inconsistently classified as de-escalation
Dose reduction	Generally is not considered de-escalation unless accompanied by spectrum modification

**Table 3:** Approaches to antibiotic-spectrum ranking and their application in defining de-escalation.

Study	Ranking Approach	Application
Trupka et al., 2017 [38]	Gram-negative agents were ranked by spectrum, from carbapenems as the broadest to ceftriaxone as the narrowest	A switch to a lower-ranked regimen, reduction in antibiotic number, or discontinuation of a pathogen-specific component was classified as de-escalation
De Bus et al., 2020—DIANA study [18]	A previously validated antibiotic-spectrum ranking was used to assess β-lactam transitions	Ninety-one percent of β-lactam changes classified as de-escalation were concordant with the ranking; common transitions included piperacillin–tazobactam to a third-generation cephalosporin or narrower penicillin/β-lactamase inhibitor
Roper et al., 2023 [33]	Pivotal Gram-negative antibiotics were allocated to predefined spectrum groups	Movement to a lower group was classified as pivotal de-escalation; an example was meropenem to cefepime
Aldardeer et al., 2023 [35]	Antipseudomonal agents were ranked according to spectrum	De-escalation required replacement of the principal antipseudomonal antibiotic with a lower-ranked agent; discontinuing MRSA coverage or one component of double antipseudomonal therapy alone was not counted
Aissaoui et al., 2025 [32]	β-lactams were classified into six groups according to spectrum and presumed ecological impact	Treatment changes following pneumonia multiplex PCR were classified as de-escalation, escalation, or other modification; results were not reported by individual β-lactam
Nikolai et al., 2026 [52]	An antibiotic hierarchy based on WHO AWaRe categories and published spectrum rankings was used	De-escalation included transition to a lower-ranked active agent or reduction from combination therapy to monotherapy by day 5
Lakbar et al., 2020 [4]	Systematic review describing institutional spectrum and ecological rankings	Highlighted heterogeneity between definitions and the absence of a universally accepted ranking system

**Table 4:** Reported de-escalation rates by study population and clinical context.

Study	Population or Intervention	De-Escalation Finding
Souza-Oliveira et al. [31]	Ventilator-associated pneumonia	Approximately 10%
Aissaoui et al. [32]	Mechanically ventilated pneumonia; multiplex PCR	11% de-escalation or cessation
De Bus et al. [18]	DIANA multinational ICU cohort	16.1% by day 3
Kim et al. [19]	Antibiotic use after withdrawal of life-sustaining treatment	17.0%
Roper et al. [33]	Culture-negative ICU infection	22.0% pivotal-agent de-escalation
Ali et al. [26]	Non-adherent carbapenem prescriptions	25.4%
Lakbar et al. [21]	ICU patients during COVID-19	33.3% among empirical treatments; de-escalation was less frequent during the COVID-19 period than in the historical control cohort (27.6% vs. 52.2%, *p* < 0.001) *
Salahuddin et al. [47]	Critically ill patients receiving empirical treatment	Approximately 48%
Trupka et al. [38]	Clinically eligible mechanically ventilated patients	67.3% with early antimicrobial de-escalation versus 66.0% with routine antimicrobial management; *p* = 0.845 *
Nikolai et al. [52]	Extractable ICU subgroup	71.0% in ICU patients versus 52.8% in non-ICU patients; ICU treatment independently favored de-escalation (adjusted OR for non-de-escalation 0.60, 95% CI 0.38–0.95; *p* = 0.029 *)

The de-escalation rates are descriptive proportions, unless a comparative statistical analysis is explicitly reported. * Statistically significant comparative finding, as reported in the original publication (*p* < 0.05 or 95% confidence interval excluding the null value). Statistical significance was not inferred for studies reporting only a single de-escalation proportion.

**Table 6:** Mortality outcomes reported across direct de-escalation comparisons, stewardship interventions, and secondary evidence sources.

Study	Mortality Outcome
De Bus et al. [18]	28-day mortality: 15.8% with de-escalation vs. 19.4% without de-escalation; not significant
Aldardeer et al. [35]	ICU mortality: 33.6% vs. 40.0%; hospital mortality: 39.2% vs. 45.6%; neither significant
Roper et al. [33]	ICU mortality: 7.9% vs. 15.6%; hospital mortality: 15.8% vs. 19.3%; neither significant
Choudhuri et al. [40]	ICU mortality: 7.3% after earlier vs. 11.4% after late de-escalation; not significant
Le et al. [36]	Mortality: 16.1% after successful discontinuation vs. 25.0% with treatment failure; not significant
Ali et al. [26]	30-day mortality: 11.1% when stewardship recommendations were accepted vs. 11.8% when rejected
Gu et al. [29]	Mortality: 19.6% before vs. 22.6% after pharmacist intervention; not significant
Lakbar et al. [4], systematic review	RCT: 31% vs. 23%, *p* = 0.55; observational synthesis favored de-escalation but was susceptible to bias
Tanzarella et al. [17], review	Pooled mortality RR 0.68, 95% CI 0.52–0.88

**Table 7:** Clinical, microbiological, and safety outcomes of antibiotic de-escalation by outcome domain.

Outcome Domain	Evidence Synthesis
Clinical cure/clinical response [18,26,37,39,43]	Generally similar or higher after de-escalation; DIANA reported day-7 cure of 57.9% vs. 42.7%.
Treatment failure [36,38,43]	No consistent increase following de-escalation.
Recurrence/re-escalation [18,21,35,37,50]	Inconsistently reported; no consistent significant difference.
Superinfection [18,35,38,40]	Usually similar, although one randomized study reported an increase.
Microbiological eradication	Rarely reported as a separate outcome. No included empirical study provided a robust comparative analysis specifically according to de-escalation status.
MDR emergence [18,21,33,38,40,44,45,48]	No consistent significant difference.
Acute kidney injury [33]	Lower following de-escalation in one culture-negative ICU cohort.
C. difficile infection [30,33,44,54]	No significant increase in the studies reporting this outcome.

**Table 8:** Determinants of de-escalation.

Factors Favoring De-Escalation	Barriers to De-Escalation
Appropriate initial empirical treatment	Septic shock or hemodynamic instability
Clinical and hemodynamic improvement	Worsening organ dysfunction
Reliable culture and susceptibility results	Negative, unreliable, or unavailable cultures
Identification of a susceptible organism	MDR or extensively drug-resistant organisms
Negative high-quality cultures	Polymicrobial infection
High-negative-predictive-value molecular tests	Uncontrolled or uncertain infection source
Absence of resistance determinants	Absence of a narrower active alternative
Adequate source control	Previous antibiotic exposure
Monomicrobial infection	Concurrent infectious foci
Availability of a narrower active agent	Concern about molecular-test false-positive results
Daily multidisciplinary reassessment	Clinician concern about treatment failure
Pharmacist and microbiologist participation	Insufficient stewardship personnel
Formal 48–72 h antibiotic time-out	Lack of institutional protocols
Local antibiograms and decision support	Delayed communication of microbiological results

**Table 9:** From evidence to practice: proposed local recommendations, implementation steps, and resource requirements for ICU antibiotic de-escalation.

Evidence-Derived Finding	Proposed Local Recommendation	Practical Implementation	Resource Requirement	Supporting Evidences
Appropriate initial treatment is a prerequisite for safe de-escalation	Preserve adequate empirical coverage at treatment initiation, particularly in septic shock or patients at high risk of MDR infection	Develop empirical protocols according to infection source, previous colonization, recent antibiotics, and the ICU antibiogram	Low–moderate	De Bus et al. [18]; Trupka et al. [38]; Ghosh et al. [39]; Arulappen et al. [37]
Reliable microbiological information facilitates de-escalation	Obtain appropriate cultures before antibiotic administration whenever this does not delay urgent treatment	At least two blood-culture sets for sepsis; respiratory samples for HAP/VAP; urine, drainage or operative samples according to source	Low	De Bus et al. [18]; Roper et al. [33]; Zhu et al. [30]; Sellers et al. [51]
Reassessment was most commonly undertaken at 48–72 h	Introduce a mandatory antibiotic time-out at 48–72 h	Include a structured antibiotic-review field in the ICU daily chart or electronic record	Low	Panditrao et al. [27]; Mishima et al. [23]; Roper et al. [33]; De Bus et al. [18]; Aldardeer et al. [35]
Daily multidisciplinary review improves stewardship implementation	Review broad-spectrum antibiotics during daily ICU rounds	Minimum participants: intensivist and microbiology/infectious-disease representative; pharmacist involvement when available	Low–moderate	Trupka et al. [38]; Ali et al. [26]; Gu et al. [29]; Mishima et al. [23]
Results should be acted upon promptly	Make a definitive treatment decision within 24 h of obtaining reliable susceptibility results	Laboratory notification of critical cultures and MDR organisms; designated clinician responsible for documenting the decision	Low	Aldardeer et al. [35]; Moniz et al. [61]; Matuszak et al. [63]; Micek et al. [64]
Negative cultures may support de-escalation when the patient is improving	Permit narrowing or cessation despite negative cultures when adequate samples were obtained, the patient is stable, and no uncontrolled source exists	Use a culture-negative infection checklist incorporating hemodynamics, SOFA trajectory, biomarkers, imaging and source control	Low	Roper et al. [33]; Le et al. [36]; Sellers et al. [51]
Source control and clinical stability are important prerequisites	Do not evaluate antibiotics in isolation from source control	Incorporate drainage, device removal, surgical review and exclusion of another infectious focus into the time-out	Low	De Bus et al. [18]; Ghosh et al. [39]; Arulappen et al. [37]
Rapid diagnostics shorten pathogen-identification time but do not automatically produce de-escalation	Use multiplex PCR selectively in high-risk pneumonia or sepsis and link every result to a stewardship recommendation	Prioritize mechanically ventilated patients with suspected HAP/VAP, severe immunosuppression or previous antibiotic exposure	High	Miller et al. [25]; Aissaoui et al. [32]; Rodríguez-Gómez et al. [34]; Contier et al. [41]; Zhu et al. [30]
Carbapenem exposure was reduced by stewardship interventions without a consistent mortality penalty	Establish carbapenem pre-authorization or prospective review within 24–48 h	Require documentation of indication, MDR risk, cultures, planned reassessment date, and available carbapenem-sparing options	Low–moderate	Sekandarzad et al. [20]; Ali et al. [26]; Gu et al. [29]; Gardner et al. [54]
Colistin and tigecycline evidence is limited, but toxicity and restricted indications justify closer oversight	Subject colistin and tigecycline prescriptions to mandatory specialist review	Review indication, organism, susceptibility, infection site, organ function, and availability of a safer active agent	Low	Jover-Sáenz et al. [24]; Panditrao et al. [27]; Yu et al. [22]
De-escalation did not consistently increase mortality, relapse or resistance	Monitor safety outcomes locally rather than assuming that reduced antibiotic consumption represents success	Measure mortality, recurrence, re-escalation, new infection, AKI, C. difficile infection and MDR acquisition	Low–moderate	De Bus et al. [18]; Roper et al. [33]; Aldardeer et al. [35]; Choudhuri et al. [40]
Dedicated stewardship staffing may be unavailable	Begin with a small functional ICU stewardship team	ICU physician, infectious-disease physician, or microbiologist, clinical pharmacist lead, and where available, infection-control practitioner and data support	Low–moderate	Panditrao et al. [27]; Gu et al. [29]; Mishima et al. [23]

**Table 10:** Class-specific recommendations.

Antibiotic Class	When De-Escalation Should Be Considered	Preferred Action	Situations Requiring Caution
Carbapenems	Susceptible pathogen identified; clinical improvement; source controlled; active carbapenem-sparing option available	Replace meropenem or imipenem with the narrowest clinically appropriate active β-lactam; discontinue unnecessary companion therapy	Septic shock with ongoing instability, uncertain source control, polymicrobial infection, ESBL/AmpC, or carbapenemase phenotype without a reliable alternative
Cephalosporins	Broad antipseudomonal or fourth-generation cephalosporin no longer required	Transition to a narrower cephalosporin or another targeted β-lactam according to susceptibility and infection site	Risk of inducible resistance, high-inoculum infection, CNS infection, inadequate tissue penetration, or uncertain susceptibility
Colistin	A less toxic active agent becomes available, or initial suspicion of highly resistant infection is not confirmed	Discontinue colistin promptly or replace it with a microbiologically active, safer agent	Confirmed MDR infection without a reliable alternative, unstable patient, uncertain susceptibility, and inadequate source control
Tigecycline	A more targeted active treatment becomes available, or the infection no longer requires its broad tissue activity	Replace with a narrower active agent and discontinue unnecessary combination components	Infection site, bloodstream involvement, organism susceptibility, severity, and availability of alternative agents must be considered

**Table 11:** Suggested indicators for local audit of an ICU de-escalation program, grouped by domain.

Domain	Proposed Indicator
Process	Proportion of broad-spectrum prescriptions reviewed within 72 h
Process	Proportion with appropriate cultures obtained before antibiotics
Process	Proportion with a documented indication, review date, and planned duration
De-escalation	Number de-escalated divided by the number clinically eligible for de-escalation
Timing	Median time from susceptibility result to treatment modification
Consumption	DOT/1000 ICU patient-days for carbapenems, cephalosporins, colistin, and tigecycline
Consumption	Broad-spectrum DOT and total antibiotic DOT reported separately
Safety	ICU, hospital and 28-day mortality
Safety	Re-escalation within 48–72 h
Safety	Relapse, recurrent infection, and new hospital-acquired infection
Toxicity	Acute kidney injury during colistin or combination therapy
Ecological outcomes	New MDR colonization or infection and C. difficile infection
Resource outcomes	ICU length of stay and antibiotic expenditure
Diagnostic performance	Proportion of rapid-test results followed by an appropriate treatment modification

## Data Availability

The data are contained within the article or the Supplementary Material. The original contributions presented in this study are included in the article/Supplementary Material. Further inquiries can be directed to the corresponding authors.

## Supplementary Materials

The following supporting information can be downloaded at: https://www.mdpi.com/article/10.3390/antibiotics15090912/s1. PRISMA 2020 Checklist. Reference [84] is cited in the Supplementary Materials.
