# Supplementary material for: De-Escalation of Broad-Spectrum and Last-Resort Antibiotics in Critically Ill Adults with Gram-Negative Infections: A Scoping Review and Evidence-Informed Framework for Tertiary-Care ICUs
Source: Antibiotics (Basel). 2026 Sep 16;15(9):912. doi: 10.3390/antibiotics15090912 (PMC13603625; doi:10.3390/antibiotics15090912)
Supplement: Supplementary file 1 [file antibiotics-15-00912-s001.zip › antibiotics-4543386-supplementary.pdf]

PRISMA 2020 Checklist

Section and Topic	Item #	Checklist item	Location where item is reported
**TITLE**			
Title	1	Identify the report as a systematic review.	Title page, p. 1. The title describes the article as 'A Review' but the Methods section specifies the scoping design.
**ABSTRACT**			
Abstract	2	See the PRISMA 2020 for Abstracts checklist.	Structured Abstract: Background, Methods, Results, Conclusions; includes sources, dates, study count, synthesis, and implications(p. 2).
**INTRODUCTION**			
Rationale	3	Describe the rationale for the review in the context of existing knowledge.	Introduction, pp. 2–3.
Objectives	4	Provide an explicit statement of the objective(s) or question(s) the review addresses.	Introduction, p. 3; Review question and PCC framework, p. 28.
**METHODS**			
Eligibility criteria	5	Specify the inclusion and exclusion criteria for the review and how studies were grouped for the syntheses.	Materials and Methods—Review question/PCC framework, Operational definition, and Eligibility criteria, pp. 28–29.
Information sources	6	Specify all databases, registers, websites, organisations, reference lists and other sources searched or consulted to identify studies. Specify the date when each source was last searched or consulted.	Reported in Figure 2, p. 30 (Web of Science; 1 January 2016–4 August 2026; limits shown).
Search strategy	7	Present the full search strategies for all databases, registers and websites, including any filters and limits used.	The complete Web of Science search string is presented in Materials and Methods (p. 26-28); search dates and filters appear in Figure 2 (p. 30).<br>("Anti-Bacterial Agents" OR "Antimicrobial Stewardship" OR antibiotic OR antimicrobial) AND (de-escalat OR deescalat OR "de escalation" OR "spectrum narrowing" OR "narrowing therapy" OR "narrower spectrum" OR "streamlining therapy" OR "streamlined therapy" OR "antibiotic time-out" OR "antibiotic timeout" OR "antimicrobial time-out" OR "antimicrobial timeout") AND ("Intensive Care Units" OR "Critical Care" OR "Critical Illness" OR ICU OR ICUs OR "intensive care" OR "critical care" OR "critically ill")
Selection process	8	Specify the methods used to decide whether a study met the inclusion criteria of the review, including how many reviewers screened each record and each report retrieved, whether they worked independently, and if applicable, details of automation tools used in the process.	Materials and Methods—Study selection, pp. 29–30.
Data collection process	9	Specify the methods used to collect data from reports, including how many reviewers collected data from each report, whether they worked independently, any processes for obtaining or confirming data from study investigators, and if applicable, details	Materials and Methods—Data charting, p. 30.

Section and Topic	Item #	Checklist item	Location where item is reported
		of automation tools used in the process.	
Data items	10a	List and define all outcomes for which data were sought. Specify whether all results that were compatible with each outcome domain in each study were sought (e.g. for all measures, time points, analyses), and if not, the methods used to decide which results to collect.	Materials and Methods—Eligibility criteria (outcome domains), p. 29; Data charting, p. 30.
	10b	List and define all other variables for which data were sought (e.g. participant and intervention characteristics, funding sources). Describe any assumptions made about any missing or unclear information.	Materials and Methods—Review question/PCC framework and Eligibility criteria, pp. 28–29; Data charting, p. 30. No explicit assumptions for missing or unclear information are reported.
Study risk of bias assessment	11	Specify the methods used to assess risk of bias in the included studies, including details of the tool(s) used, how many reviewers assessed each study and whether they worked independently, and if applicable, details of automation tools used in the process.	No formal risk-of-bias assessment was performed because this scoping review was designed to map heterogeneous evidence rather than estimate a pooled intervention effect or formally grade comparative effectiveness. Methodological characteristics and limitations were charted for each source and considered narratively during interpretation.
Effect measures	12	Specify for each outcome the effect measure(s) (e.g. risk ratio, mean difference) used in the synthesis or presentation of results.	Materials and Methods—Evidence synthesis, pp. 30–31: numerical outcomes were reported using effect estimates supplied by the original studies; no common effect measure was imposed.
Synthesis methods	13a	Describe the processes used to decide which studies were eligible for each synthesis (e.g. tabulating the study intervention characteristics and comparing against the planned groups for each synthesis (item #5)).	Materials and Methods—Eligibility criteria, pp. 28–29; Evidence synthesis, pp. 30–31. Empirical and contextual evidence were summarized separately (Results, pp. 3–15).
	13b	Describe any methods required to prepare the data for presentation or synthesis, such as handling of missing summary statistics, or data conversions.	Not applicable. No data conversions, imputations, or preparation of missing summary statistics are reported.
	13c	Describe any methods used to tabulate or visually display results of individual studies and syntheses.	Materials and Methods—Data charting and Evidence synthesis, pp. 30–31; results displayed in Tables 1–9 and Figures 1–2, pp. 3–25 and 30.
	13d	Describe any methods used to synthesize results and provide a rationale for the choice(s). If meta-	Materials and Methods—Evidence synthesis, pp. 30–31. Descriptive and narrative synthesis was selected because of heterogeneity; no meta-analysis was performed.

Section and Topic	Item #	Checklist item	Location where item is reported
		analysis was performed, describe the model(s), method(s) to identify the presence and extent of statistical heterogeneity, and software package(s) used.	
	13e	Describe any methods used to explore possible causes of heterogeneity among study results (e.g. subgroup analysis, meta-regression).	No formal subgroup analysis or meta-regression. Heterogeneity was explored descriptively by definition, timing, antibiotic class, population, and outcome (Results, pp. 15–21; Discussion, pp. 25–27).
	13f	Describe any sensitivity analyses conducted to assess robustness of the synthesized results.	Not applicable. No sensitivity analyses were conducted.
Reporting bias assessment	14	Describe any methods used to assess risk of bias due to missing results in a synthesis (arising from reporting biases).	Not applicable/not assessed. No formal reporting-bias assessment was performed.
Certainty assessment	15	Describe any methods used to assess certainty (or confidence) in the body of evidence for an outcome.	Not applicable/not assessed. No formal certainty-of-evidence method was applied; evidence limitations are discussed qualitatively (Discussion—Limitations, p. 27).
**RESULTS**			
Study selection	16a	Describe the results of the search and selection process, from the number of records identified in the search to the number of studies included in the review, ideally using a flow diagram.	Results—Characteristics of included evidence, p. 3; PRISMA flow diagram (Figure 2), p. 30.
	16b	Cite studies that might appear to meet the inclusion criteria, but which were excluded, and explain why they were excluded.	Partially reported: aggregate reasons for full-text exclusion are shown in Figure 2 (p. 30), but individual apparently eligible excluded reports are not cited in the manuscript.
Study characteristics	17	Cite each included study and present its characteristics.	Table 1, pp. 3–15; accompanying Characteristics of included evidence, pp. 3 and 15.
Risk of bias in studies	18	Present assessments of risk of bias for each included study.	Not applicable/not reported. No formal risk-of-bias assessment was performed.
Results of individual studies	19	For all outcomes, present, for each study: (a) summary statistics for each group (where appropriate) and (b) an effect estimate and its precision (e.g. confidence/credible interval), ideally using structured tables or plots.	Table 1, pp. 3–15; Tables 4–7 and accompanying text, pp. 17–20. Effect estimates and precision are reproduced as reported in the original studies.
Results of syntheses	20a	For each synthesis, briefly summarise the characteristics and risk of bias among contributing studies.	Results, pp. 3–21, particularly Table 1 and the sections on definitions, timing, antibiotic class, mortality, safety, antibiotic exposure, and determinants. No formal risk-of-bias synthesis was performed.
	20b	Present results of all statistical	Not applicable. No statistical synthesis or meta-analysis was conducted.

Section and Topic	Item #	Checklist item	Location where item is reported
		syntheses conducted. If meta-analysis was done, present for each the summary estimate and its precision (e.g. confidence/credible interval) and measures of statistical heterogeneity. If comparing groups, describe the direction of the effect.	
	20c	Present results of all investigations of possible causes of heterogeneity among study results.	Descriptive findings on heterogeneity are reported in Results, pp. 15–21, and interpreted in Discussion, pp. 25–27; no formal heterogeneity analysis was conducted.
	20d	Present results of all sensitivity analyses conducted to assess the robustness of the synthesized results.	Not applicable. No sensitivity analyses were conducted.
Reporting biases	21	Present assessments of risk of bias due to missing results (arising from reporting biases) for each synthesis assessed.	Not applicable/not assessed. No formal reporting-bias assessment was performed.
Certainty of evidence	22	Present assessments of certainty (or confidence) in the body of evidence for each outcome assessed.	Not applicable/not assessed. No formal certainty-of-evidence assessment was performed.
**DISCUSSION**			
Discussion	23a	Provide a general interpretation of the results in the context of other evidence.	Discussion, pp. 25–27.
	23b	Discuss any limitations of the evidence included in the review.	Discussion—limitations of the evidence, p. 27.
	23c	Discuss any limitations of the review processes used.	Discussion—Limitations, p. 27 (English-language and date restrictions); other review-process limitations are not discussed in detail.
	23d	Discuss implications of the results for practice, policy, and future research.	Results—Romanian tertiary-ICU framework, Tables 9–10 and Figures 1–2, pp. 21–25; Discussion and Future directions, pp. 25–27; Conclusions, p. 31.
**OTHER INFORMATION**			
Registration and protocol	24a	Provide registration information for the review, including register name and registration number, or state that the review was not registered.	Materials and Methods—Review design and reporting framework, p. 27: PROSPERO CRD420261478424.
	24b	Indicate where the review protocol can be accessed, or state that a protocol was not prepared.	Partially reported. A protocol is stated to have been developed and registered (p. 27), but a direct protocol location or access link is not provided.
	24c	Describe and explain any amendments to information provided at registration or in the protocol.	Not reported. No amendments to the protocol or registration are described.
Support	25	Describe sources of financial or non-financial support for the review, and the role of the funders or sponsors in	Not reported. The Funding field is blank (p. 32).

Section and Topic	Item #	Checklist item	Location where item is reported
		the review.	
Competing interests	26	Declare any competing interests of review authors.	Not adequately reported. The Conflicts of Interest field contains only 'D' (p. 32).
Availability of data, code and other materials	27	Report which of the following are publicly available and where they can be found: template data collection forms; data extracted from included studies; data used for all analyses; analytic code; any other materials used in the review.	Not reported. The Data Availability Statement field is blank (p. 32).

Page, M.J.; McKenzie, J.E.; Bossuyt, P.M.; Boutron, I.; Hoffmann, T.C.; Mulrow, C.D.; Shamseer, L.; Tetzlaff, J.M.; Akl, E.A.; Brennan, S.E.; et al. The PRISMA 2020 statement : an updated guideline for reporting systematic reviews. *BMJ* **2021**, *372*, n71. doi:10.1136/bmj.n71.
